# Supplementary figures and images for: Widespread misregulation of inter-species hybrid transcriptomes due to sex-specific and sex-chromosome regulatory evolution
Source: PLoS Genet. 2021 Mar 5;17(3):e1009409. doi: 10.1371/journal.pgen.1009409 (PMC7968742; doi:10.1371/journal.pgen.1009409)

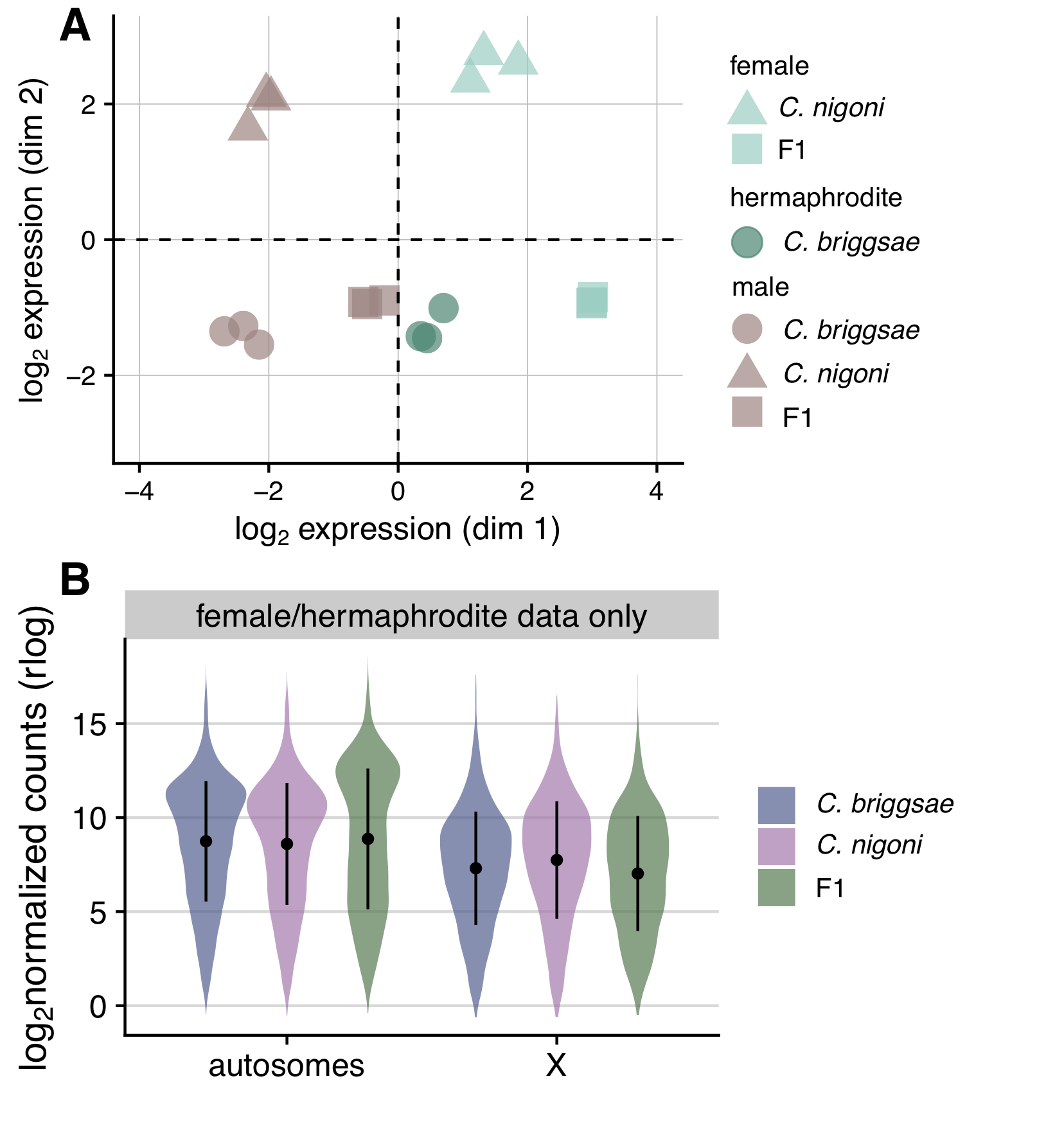

Supplement: S1 Fig — (A) Multi-dimensional scaling plot showing overall expression distance between samples. (B) magnitude of expression (rlog-transformed counts) for autosomes and the X-chromosome across C. briggsae hermaphrodites, C. nigoni females, and F1 females. The X-chromosome has, on average, lower magnitude of gene expression than autosomes, which is expected with dosage compensation. However, F1 hybrids do not show a strong pattern of over-active dosage compensation leading to generalized lower levels of expression. (TIF) [file pgen.1009409.s001.tif]

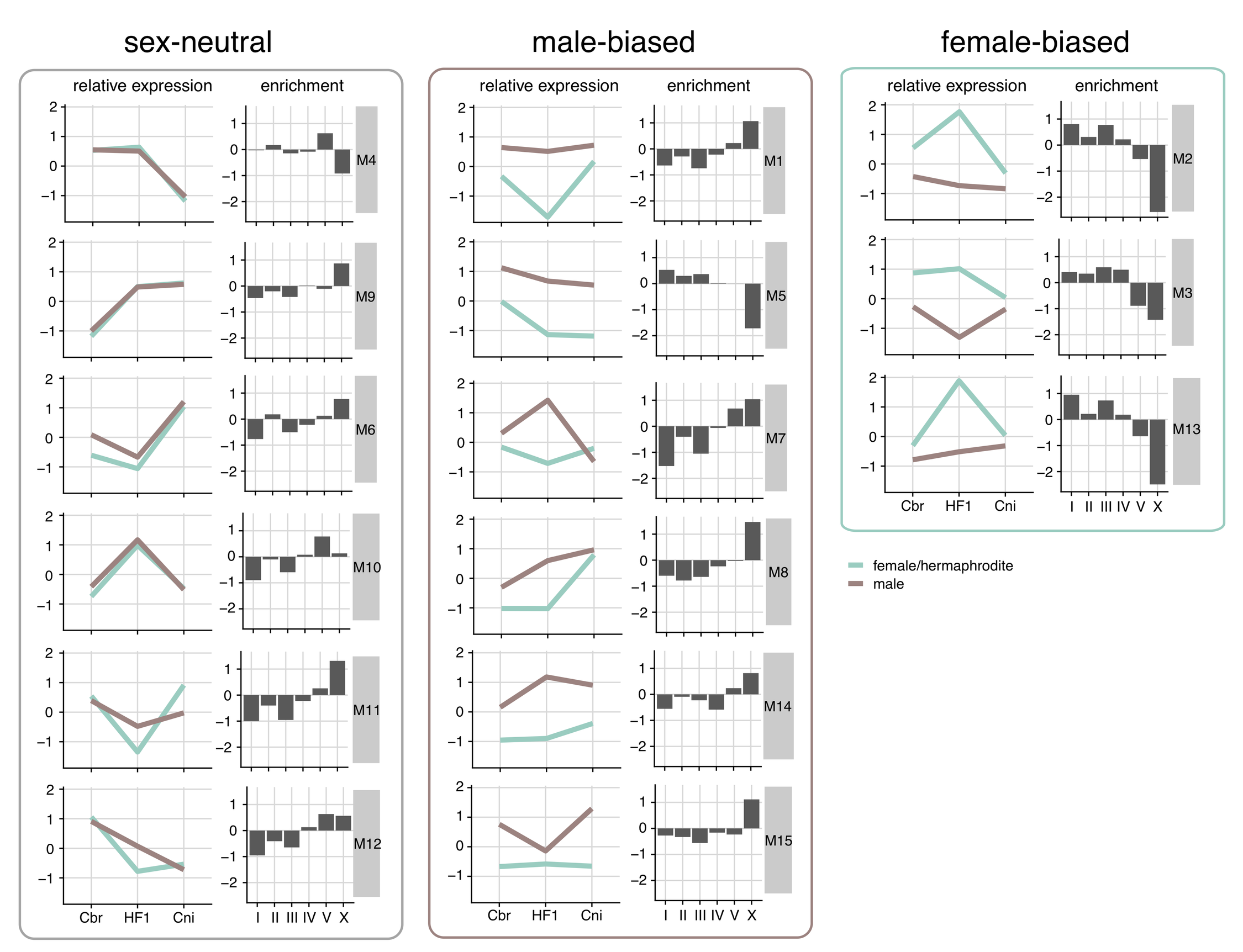

Supplement: S2 Fig — The naming scheme for each of the clusters is arbitrary. Numbers inside circles indicate the number of genes in each cluster. Enrichment/depletion within chromosomes is represented by the log2 odds ratio (i.e., observed/expected), with positive values indicating enrichment and negative values depletion. (TIF) [file pgen.1009409.s002.tif]

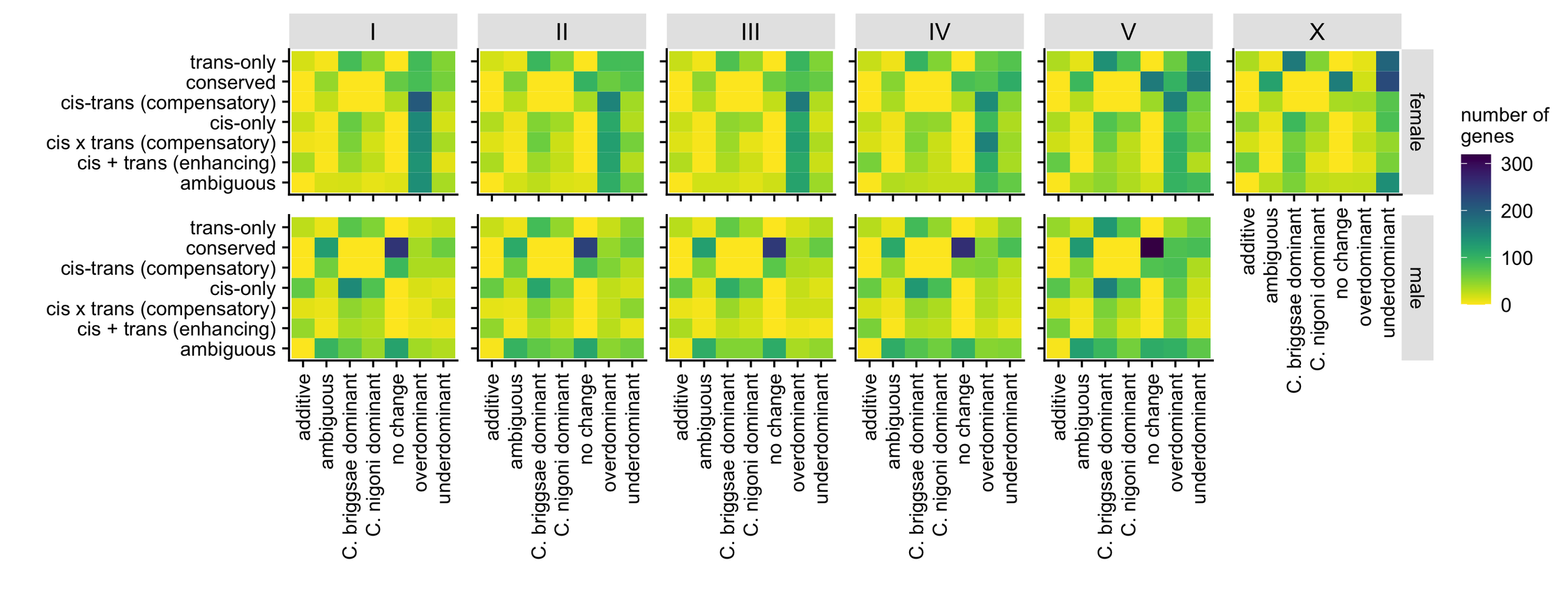

Supplement: S3 Fig — Heatmap of the number of genes in each expression inheritance group (x-axis) for each type of cis and trans regulatory changes (y-axis) for each chromosome (I-V, X) and each sex. Includes all categorizations. (TIF) [file pgen.1009409.s003.tif]

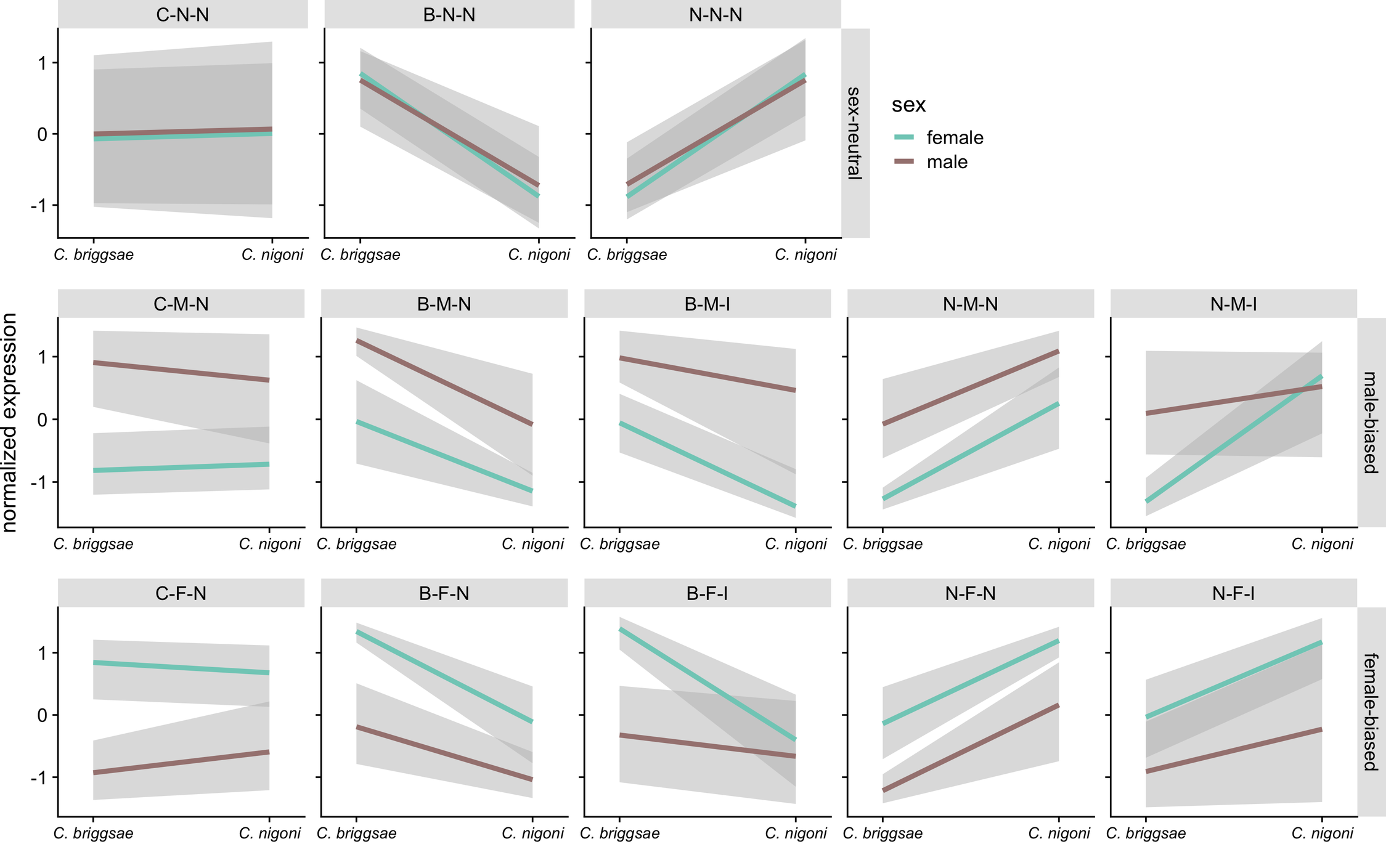

Supplement: S4 Fig — Rows show relative expression of sex-neutral, male-biased, and female-biased genes. Groups that include an "I" at the end in the name code have significant species-by-sex interactions. (TIF) [file pgen.1009409.s004.tif]

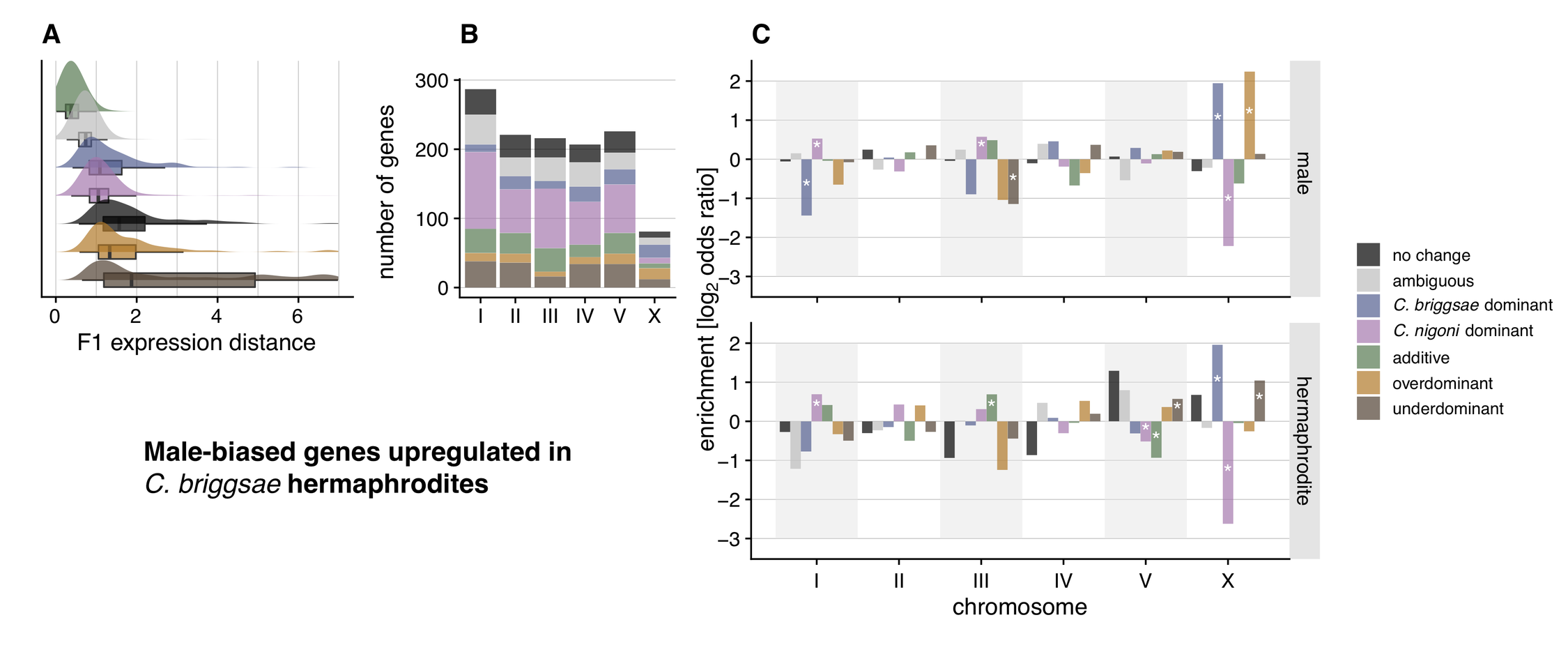

Supplement: S5 Fig — The X-chromosome is depleted compared to autosomes (B) but has distinct relative enrichments of genes with C. briggsae expression dominance in both males and "females" in contrast to autosomes, and presents different misexpression categories between males (overdominant) and females (underdominant). (TIF) [file pgen.1009409.s005.tif]

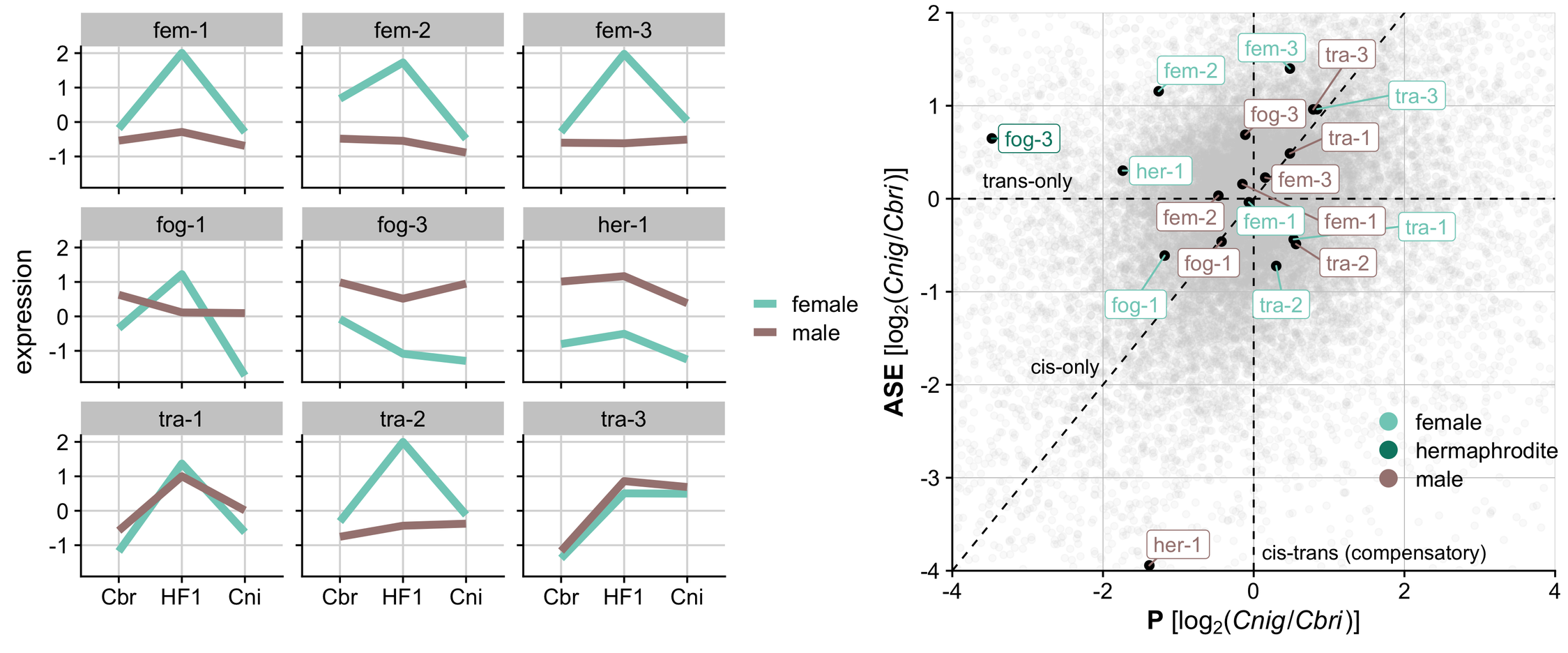

Supplement: S6 Fig — The left panel shows relative (log-transformed and normalized) expression for males (brown) and females (light green) in C. briggsae (Cbr), F1 hybrids (HF1), and C. nigoni (Cni). The right panel shows a biplot between the log-fold change in allele-specific expression (y-axis) and the log-fold change in expression divergence between species (x-axis). Dotted lines mark expected trajectories for trans-only (horizontal), cis-only (diagonal), and cis-trans compensatory (vertical) changes. Grey dots represent the regulatory space of other orthologs in our dataset. (TIF) [file pgen.1009409.s006.tif]

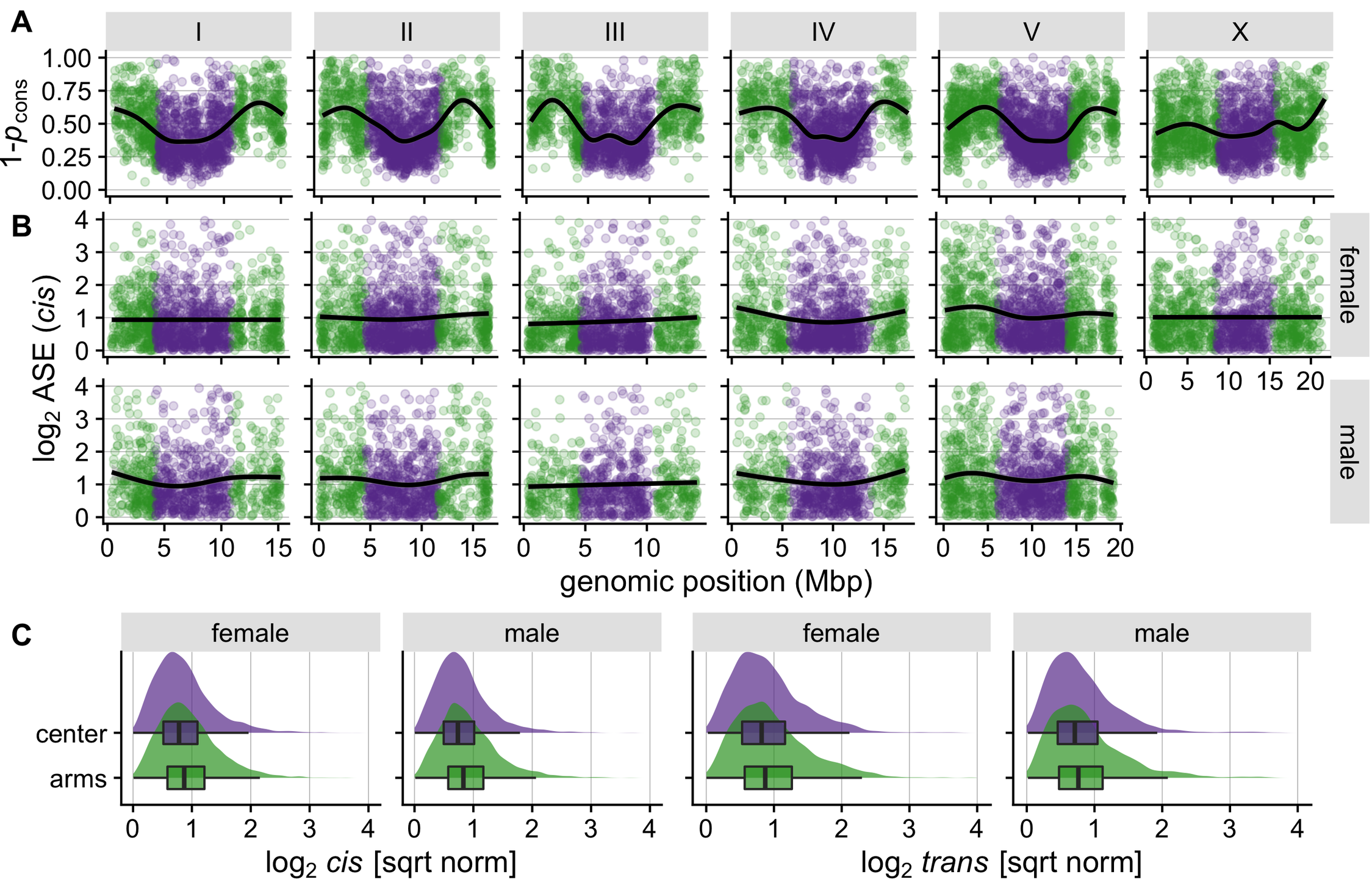

Supplement: S7 Fig — Chromosomal arm (green) and center (purple) regions differ strongly in (A) upstream sequence divergence (1-Pcons; proportion of non-conserved 5 bp windows within 500 bp upstream of each gene) but only moderately in (B) cis-regulatory divergence (log2 allele-specific expression, ASE; females on top). (A) Proportion of non-conserved 5 bp windows within 500 bp upstream of each gene (1-Pcons) for each chromosome. (B) Absolute magnitude of log2 allele-specific expression or cis-regulatory divergence for each chromosome in females and for autosomes only in males. Black lines in A and B indicate general additive regression (GAM) trendlines. (C) Arm and center regions do not differ greatly in magnitude of regulatory divergence. Box- and density plots of total (absolute) cis-regulatory divergence (left two panels) and trans regulatory divergence (right two panels) for either male or female gene expression. Allele-specific expression values are square-root-normalized in C to facilitate visual comparison. (TIF) [file pgen.1009409.s007.tif]

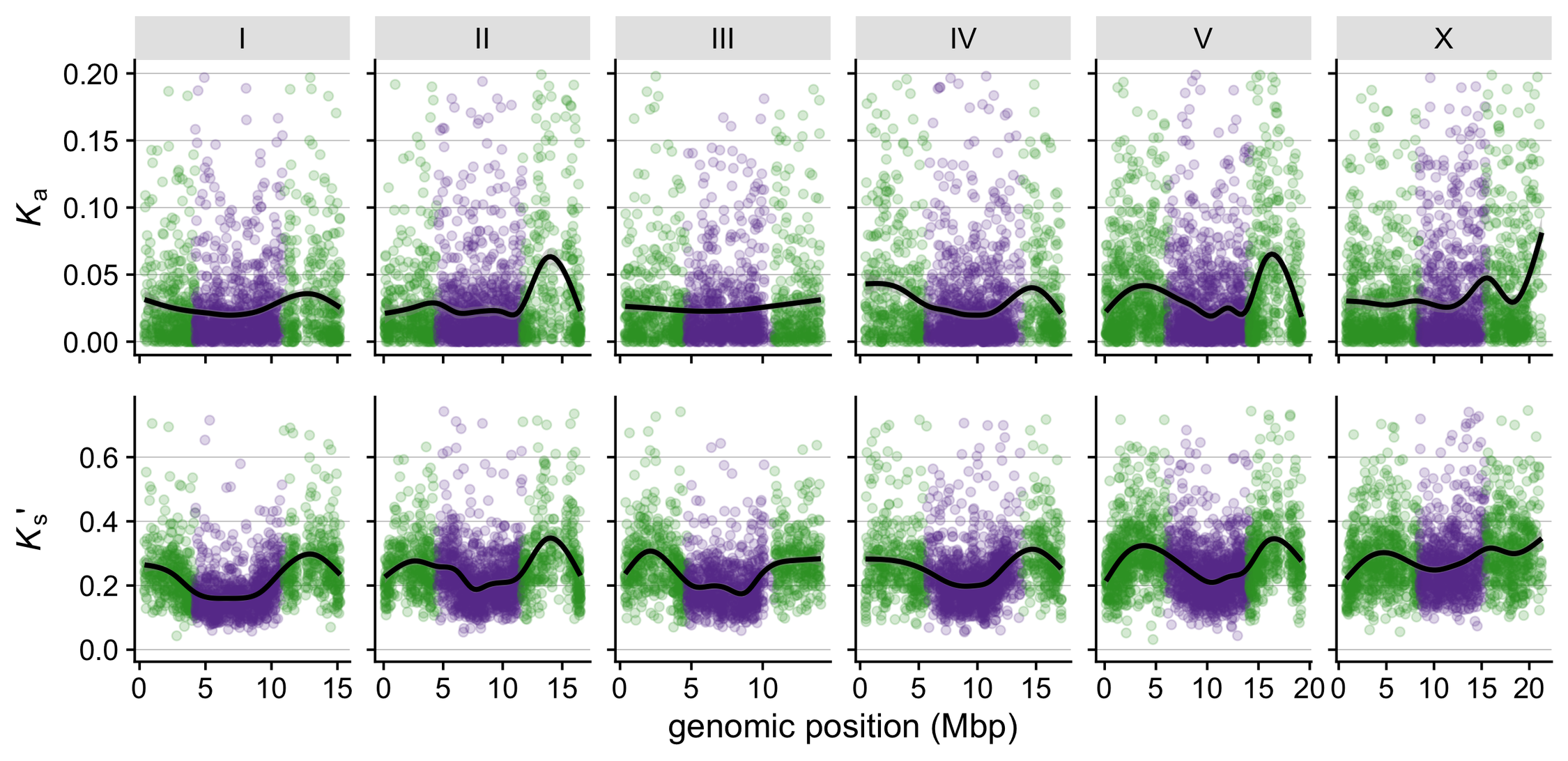

Supplement: S8 Fig — Coding sequence evolutionary rates for replacement sites (Ka) and synonymous sites (Ks’, adjusted for selection on codon usage) for 13,636 orthologs between C. briggsae and C. nigoni along the chromosome positions of the C. briggsae genome. Colors mark chromosome arms (green) and center (purple); black lines indicate general additive regression (GAM) trends. (TIF) [file pgen.1009409.s008.tif]

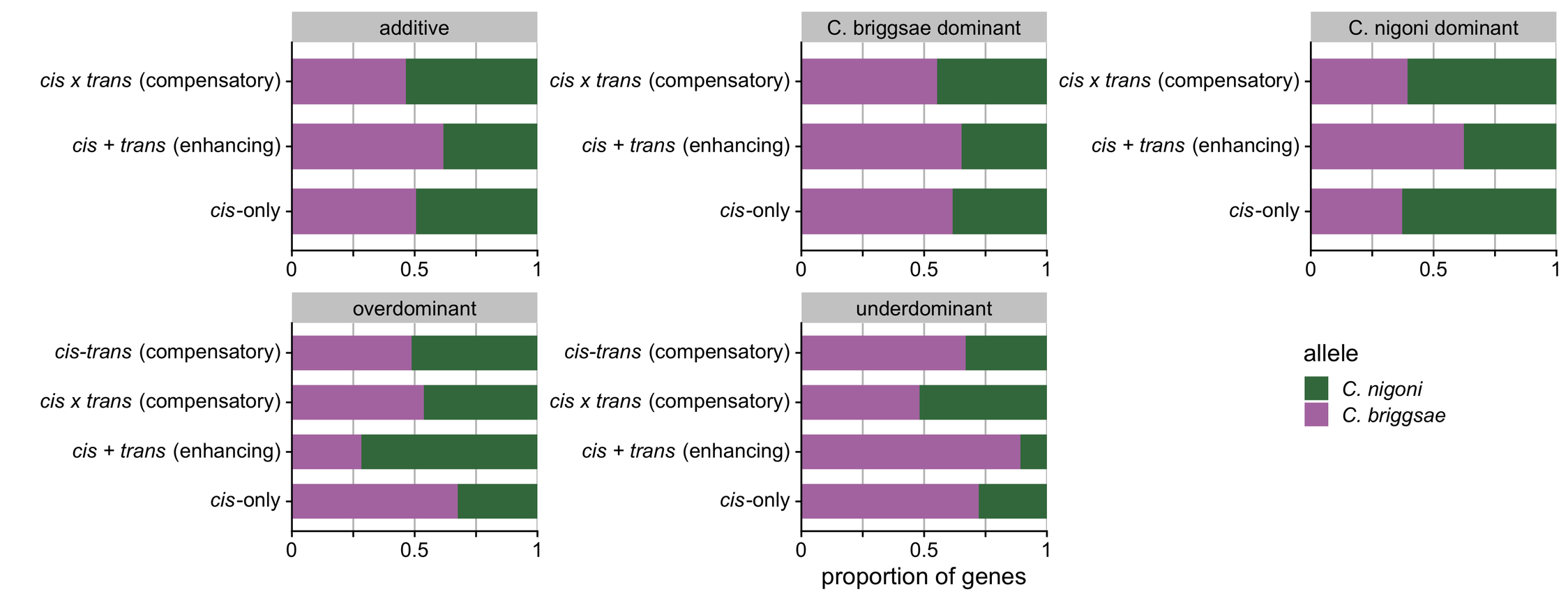

Supplement: S9 Fig — Proportion of autosomal genes with significant allele-specific expression (y-axis) showing either additive expression (first panel, top row), simple expression dominance of one species (second and third panels, top row) and transgressive over- or under-dominant expression (bottom panels) in hybrid males. Genes with conserved regulation or trans-only effects were excluded for not having significant allele-specific expression. (TIF) [file pgen.1009409.s009.tif]

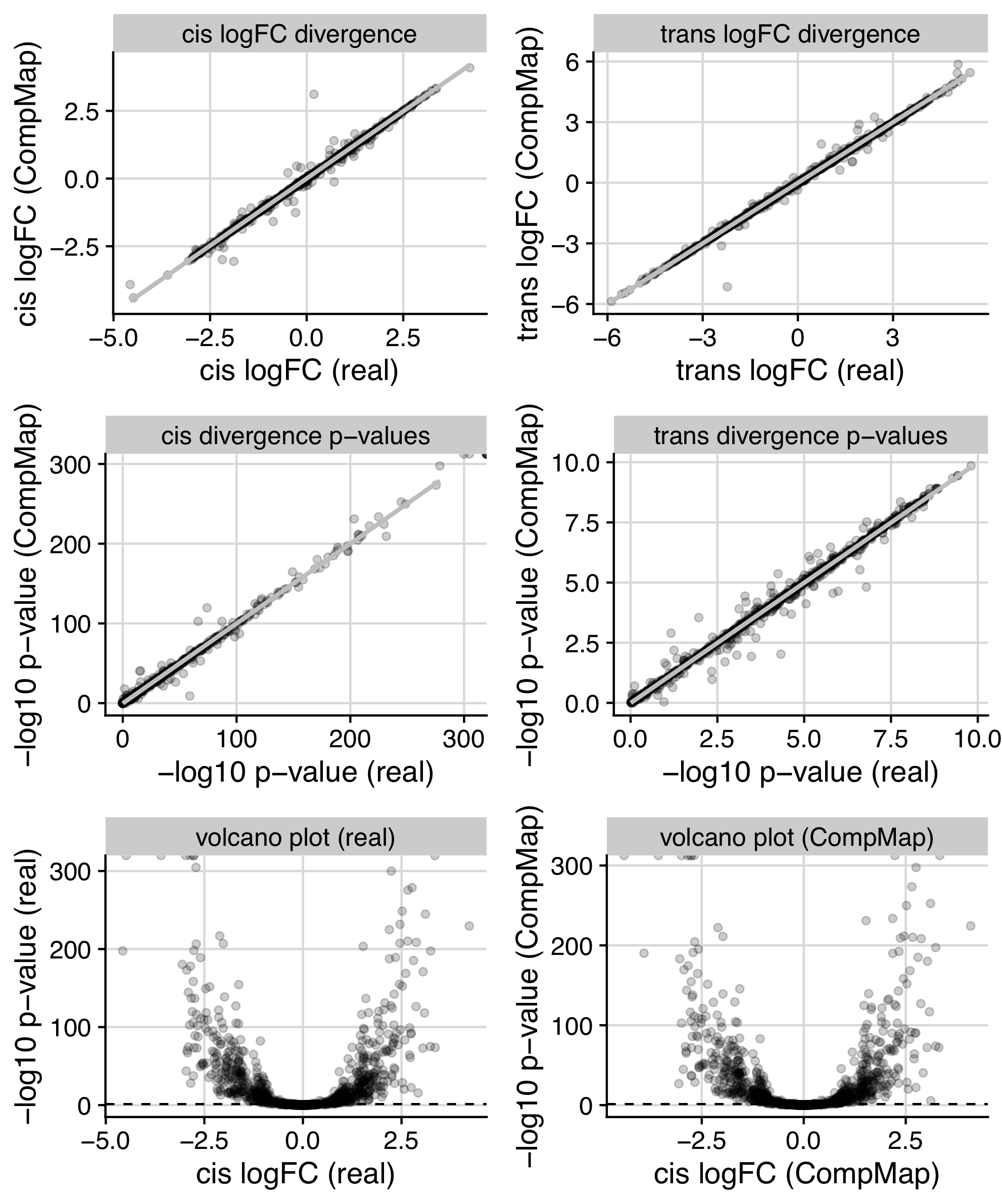

Supplement: S10 Fig — Scatter plot and linear regressions (top 4 panels) between regulatory divergence (log-fold-change) due to allele-specific expression or cis changes and their FDR corrected P-values, regulatory divergence due to trans effects and their FDR corrected P-values, and volcano plots (lower panels) comparing results from simulated RNA-seq allele-specific expression data generated by polyester and allele-specific read counts by CompMap. (TIF) [file pgen.1009409.s010.tif]

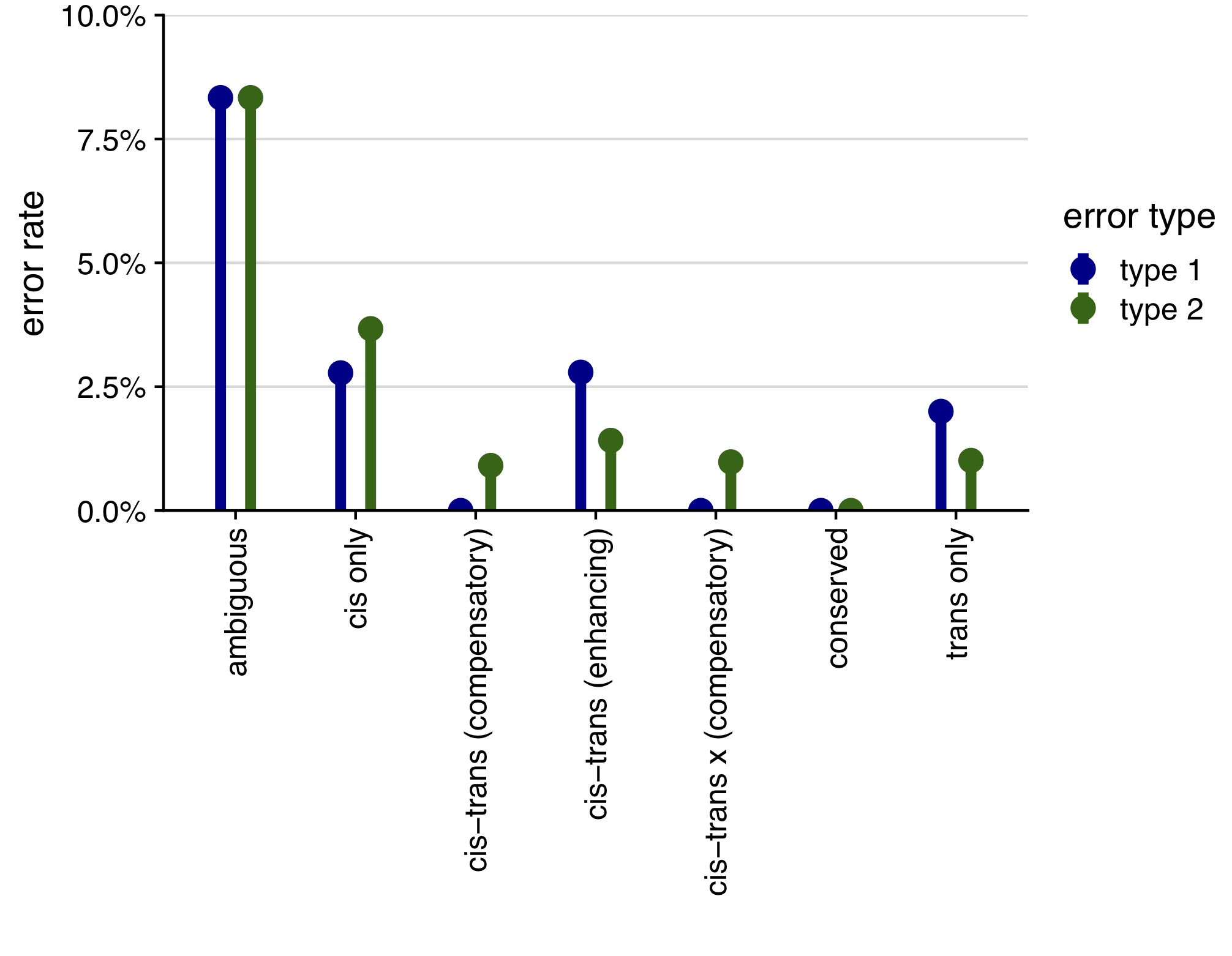

Supplement: S11 Fig — Expected values were drawn from RNA-seq allele-specific expression data simulated with polyester. Observed values were drawn based on inferences drawn from CompMap allele-specific count data. (TIF) [file pgen.1009409.s011.tif]

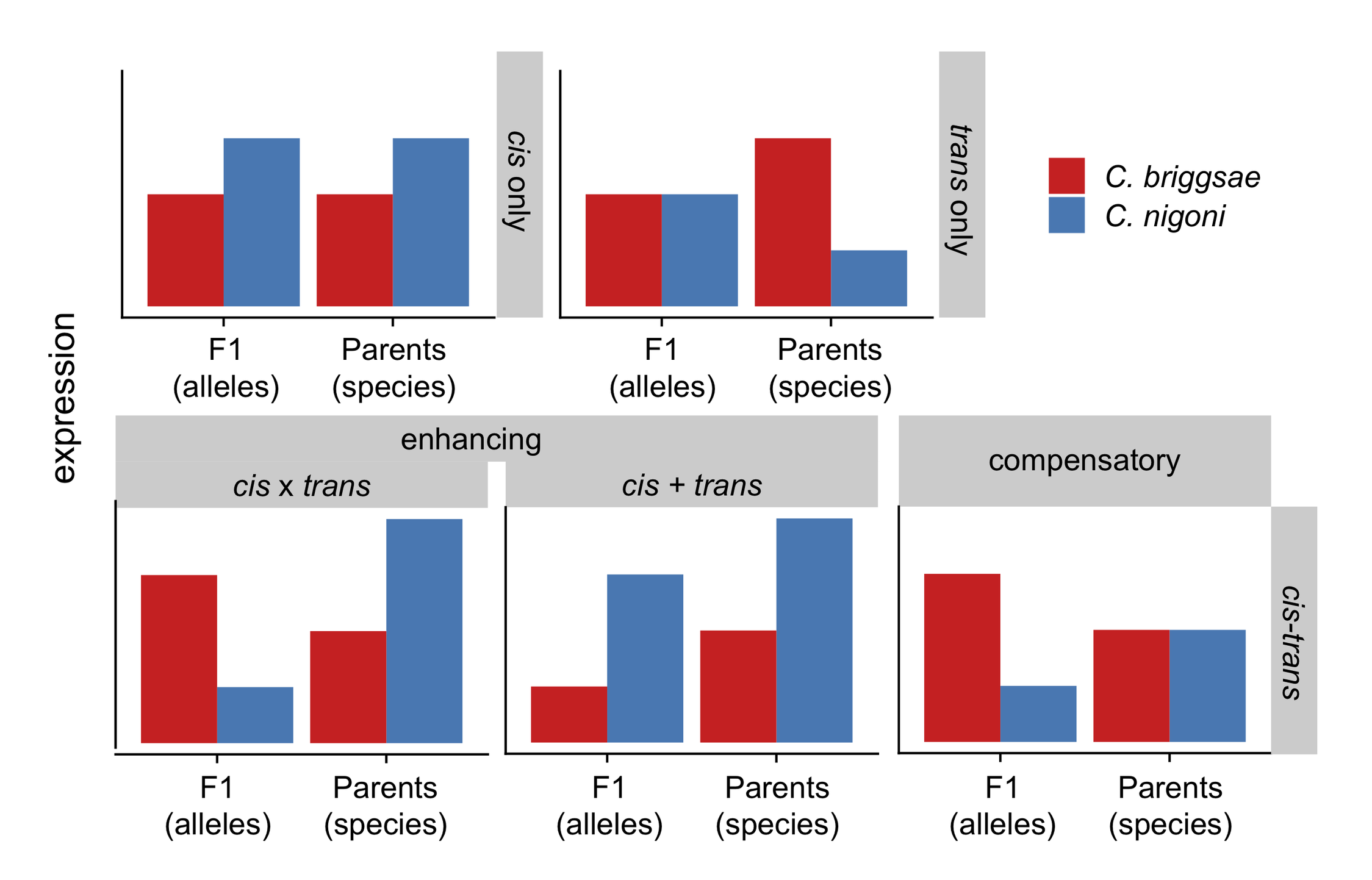

Supplement: S12 Fig — (TIF) [file pgen.1009409.s012.tif]

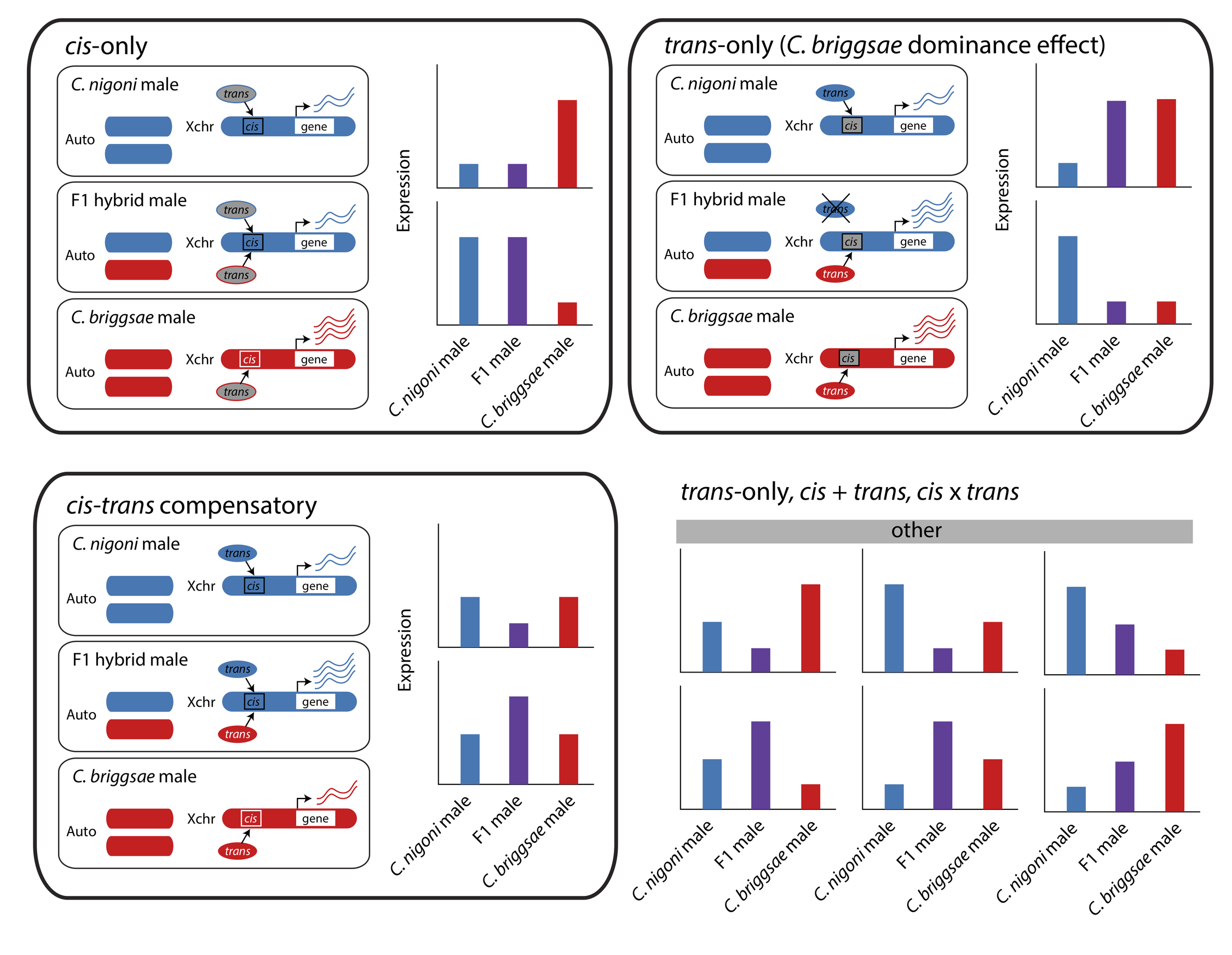

Supplement: S13 Fig — Categories not fitting into either cis-only, trans-only, or compensatory cis-trans were lumped into "other". (TIF) [file pgen.1009409.s013.tif]
