# Supplementary material for: Widespread misregulation of inter-species hybrid transcriptomes due to sex-specific and sex-chromosome regulatory evolution
Source: PLoS Genet. 2021 Mar 5;17(3):e1009409. doi: 10.1371/journal.pgen.1009409 (PMC7968742; doi:10.1371/journal.pgen.1009409)
Supplement: Attachment — Submitted filename: reviewers_comments_v3.docx [file pgen.1009409.s014.docx]

Dear Editors,

We appreciate you having solicited such thoughtful reviews of our manuscript. All comments and concerns raised by the reviewers were deeply insightful and have helped us improve our manuscript both structurally and analytically. We have now addressed, to the best of our abilities and given the constraints of our work, all of the reviewer's comments. Here we summarize the six most substantive revisions, with other improvements and modifications described in detail inline with the specific reviewer comments below:

- Reviewer #2’s comments about our competitive mapping approach led us revisit and validate our approach using simulated data, giving us greater confidence in our results. We are happy to find that the re-analysis remains largely consistent with our original findings. We have updated the manuscript accordingly, including new figures were appropriate. We are also going ahead in preparing a paper of the competitive-mapping program as a separate publication to *Bioinformatics*.
- We substantially revised our treatment of expression divergence on the X chromosome for hybrid males, and the recategorization and presentation of cis and trans divergence, in response to important points raised by all reviewers.
- We now use publicly-available RNA-seq data from C. briggsae hermaphrodites with a she-1 mutant background (conferring on hermaphrodites an effectively "female" phenotype) to better define male-biased genes that are differentially expressed between the C. briggsae “females” and hermaphrodites in order to make better calibrated comparisons for “females”. As a result, we also analyzed separately male-biased hermaphrodite-upregulated genes (Fig. 5).
- Spermatogenesis genes are now analyzed in a more consistent way and are presented in a separate figure. We also added data, results, and discussion about genes involved with the sex determination pathway.
- We addressed the issue of dosage compensation with additional analyses and reference to relevant literature.
- Raw read data are now available at NCBI under the BioProject number PRJNA659254 and all our downstream analyses, including scripts, count data, and tables are accessible through a GitHub repository (<https://github.com/santiagosnchez/competitive_mapping_workflow>).

We hope that you now find this revised manuscript suitable for publication in PLoS Genetics.

Sincerely,

Santiago Sanchez-Ramirez and Asher Cutter

Dear Dr. Sanchez-Ramirez,

Thank you very much for submitting your manuscript "Sex-specific and sex-chromosome regulatory evolution underlie widespread misregulation of inter-species hybrid transcriptomes" (PGENETICS-D-20-00671) for consideration at PLOS Genetics. As with all papers peer reviewed by the journal, your manuscript was reviewed by members of the editorial board and by several independent peer reviewers. We apologize for how long it took to review your manuscript, but I hope you will agree that the resulting reviews are very thorough.  The subject of this study is of major importance in evolutionary genetics, especially given how little work along these lines has been done in nematodes, and the fact that some of the described patterns differ from those reported from other models.  However, there are substantial concerns about data interpretation.  The two most important ones center on the effects of tissue composition and allometric differences on the interpretation of male- and
female/hermaphrodite-biased expression; and on the impact of possible X-autosome interactions on the decomposition of cis, trans, and cis*trans variation in XX and X0 hybrids, especially as it affects X-linked gene expression. Given the centrality of these concerns to the goals and conclusions of this study, we regret to inform you that we will not be pursuing this manuscript for publication at PLOS Genetics in its present form.  We would be willing to consider a thoroughly revised study that implements additional analyses to address these concerns, as well as several other substantive issues raised by the reviewers.

The reviews are attached below this email, and we hope you will find them helpful if you decide to revise the manuscript for submission elsewhere. We are sorry that we cannot be more positive on this occasion. We very much appreciate your wish to present your work in one of PLOS's Open Access publications.

Thank you for your support, and we hope that you will consider PLOS Genetics for other submissions in the future.

Sincerely,

Artyom Kopp
Associate Editor
PLOS Genetics

Bret Payseur
Section Editor: Evolution
PLOS Genetics

>>> We have done a major rehaul of our manuscript, including re-scoring allele-specific expression (CompMap and simulation validation) and read counts for each species (featureCounts), analytical approaches for differential expression (DESeq2), and re-categorizations, and separately analyzing male-biased genes upregulated in hermaphrodites. In addition to this, we have reinterpreted some of our results including cis and trans regulatory divergence, in particular those affecting the X-Chromosome in hybrid males. We also discuss morphological/anatomical differences relating to allometric issues that our data, and information from the literature, can inform. We are confident that these and other changes and improvements have addressed the helpful comments raised by the reviewers.

**************************************
Reviewer's Responses to Questions

**Reviewer #1:**

Point 1:
One large concern about the “large-X” discussion in the manuscript is that there seems to be a confusion of the genetic effect of the X-chromosome with misregulation of the genes on the X-chromosome. The genetic effect of the X-chromosome on genetic incompatibility has been revealed by using deficiency series in Drosophila (Massly and Presgraves 2007) or by using introgression lines in nematodes (Bi et al. 2015). In these cases, the genetic effects are not confined to misexpression of the genes located on the X-chromosome, but includes any incompatibility caused by X-autosome interaction (including misexpression of the genes on the autosomes caused by changes on the X chromosome). For misregulation of the genes on the X-chromosome, only the expression divergence involving cis-regulatory changes is relevant to X-autosome incompatibility since trans-only divergence is not due to mutations on the X-chromosome (assuming that the trans factors are on the autosomes). Therefore, from my point of view, enrichment of the cis-trans divergence in males (but not in females) shown in Fig. 4H is an indication of large-X effect. In this sense, I do not think the overall pattern counters expectation of the “large-X” effect (Lines 27-28). Also, while it is true that numerous X-linked hybrid male sterility factors are reported in Drosophila (Lines 509-510), male-biased expression genes are also depleted on the X-chromosome (Parisi et al. 2003 Science; Sturgill et al. 2007 Nature). Therefore, not exactly in contrast to nematodes as in Line 509-510.

Response:

>>> Thank you for raising this point and for providing additional references on this topic, which we now cite. We agree that the "large-X" effect, as described using defined using deficiency or introgression lines, is not equivalent to a widespread misregulation in a broad sense. We also agree with your suggestion about cis-regulatory changes on the X and their effects on X-autosome interactions. Following your comment, we have now updated that section of the discussion to make a clear distinction between a "large-X" effect arising from X-autosome incompatibilities and a "large-X" effect arising for misregulation. We have also updated our figures (Fig. 3) to make clearer the point you highlight about enrichment of X-linked cis-trans divergence for males providing an indication of a large-X effect.

Point 2:
The nematode sexual reproduction system needs to be explained briefly for readers like me who are not familiar with these species. It should at least be mentioned that C. briggsae are mostly selfing species with hermaphrodites (referred to as females in the manuscript) and males, whereas C. nigoni are outbreeding species with females and males. I was not sure whether the F1 hybrid female is a female or a hermaphrodite. The authors do not distinguish between females and hermaphrodites, but I did not feel comfortable categorizing genes with biased expression in hermaphrodites as female-biased genes, the same category as in a species with a real female. I suspect that male-biased genes can be underrepresented in male-biased gene category in C. briggsae. Some clarification is necessary regarding this issue. The authors actually mention in a later section about genes involved in spermatogenesis being expressed in hermaphrodites (Lines 312-314).

Response:

>>> As suggested, we have added information and explanation about the sexual system to the introduction and the methods sections, including the hybrids (they are females, not hermaphrodites). Hermaphrodites and females share the same somatic phenotype, but hermaphrodites have a gonad that transiently makes sperm cells. To improve the biological appropriateness of comparisons of the sexes, we now incorporate analysis of differential expression between C. briggsae hermaphrodites and C. briggsae she-1 mutants, which precludes spermatogenesis in hermaphrodite gonads, making them "pseudo-females" (data from Thomas et al. 2012). With this additional analysis, we identify a set of about 1,000 genes that are male-biased and also upregulated in hermaphrodites (Fig. 5). These genes are enriched for orthologs that are upregulated during spermatogenesis in C. elegans (Ma et al. 2014) and for a cluster of male-biased genes that are upregulated in C. briggsae hermaphrodites, having a species-by-sex interaction (B-M-I; Figs. 4 and 5). Thus, we interpret this group of ~1,000 genes as representing hermaphrodite-expressed genes associated with male function (i.e. spermatogenesis). In our efforts to mitigate potential biases due to differences in mating systems, we have excluded this set of genes from all analyses where C. briggsae hermaphrodites and C. nigoni females are compared.

Point 3:
I think that the divergence scoring for the X-chromosome in males (XO) shown in Supplementary Fig. S3 should be taken with caution. While this is probably the best option in the hemizygous situation, it is obviously different from the cases in other chromosomes or in females. The trans-only pattern shown in Fig. S3 assumes that the trans factor from C. nigoni is recessive to that from C. briggsae (explained in Line 736-737). I think that this assumption is arbitrary and it can be codominant, which then gives an intermediate expression level in F1 hybrid male. This pattern is shown as the right most graphs in those categorized as cis-trans (enhancing). The enrichment of the cis-trans (enhancing) genes on the X-chromosome of male shown in Fig. 2H could be partly influenced by such effect. Also, strictly speaking, trans factors located on the X chromosome that regulate the genes on the same X chromosome are ignored.

Response:

>>> Thank you for making this observation. We also had concerns about how valid it would be to compare male X-linked to females and autosomes. Correspondingly, we have updated and clarified how patterns of regulatory divergence can be quantified and inferred, albeit in a more limited fashion, for X-linked genes in hybrid males from the kind of data that we present. At least three types of regulatory divergence patterns can be confidently identified, with some limitations (Fig. 3):

- First, X-linked cis-only regulatory divergence would necessarily involve gene expression in F1 males matching expression from C. nigoni. Divergence in trans-regulatory factors would lead to deviation of this pattern, either upwards or downwards. The assumption that the paternal C. briggsae X chromosome does not contribute trans-regulatory factors is also implicit for hybrid male autosome regulatory divergence (i.e. it would be an assumption also applied to autosome genes in males). In the revised manuscript, we refer to such genes as “cis-only*”.
- Second, X-linked genes in male hybrids with expression that matches levels observed in C. briggsae males would imply that there is no significant regulatory divergence due to cis-acting factors, with the implicit assumption that any C. nigoni trans-acting factors are recessive. This partial view likely underestimates total trans-regulatory factors affecting X-linked genes, so we have decided to mark the distinction between trans-regulatory divergence for autosomes and the X for males and females (i.e., X-linked trans-regulatory changes for males involve a subset –underestimation– of those observed for autosomes in males and for both the X and autosomes for females). In the revised manuscript, we refer to such genes as “trans-only*”.
- Third, X-linked “cis-trans compensatory” changes can be identified in cases where there are no significant differences in expression between parent species despite significant deviations in expression in hybrid males. Exposure to both C. briggsae and C. nigoni trans environments would lead to such overdominant and underdominant expression among X-linked genes in hybrid males as a consequence of the evolution of compensatory factors in regulation.
- All other genes were combined into an "other" category. This logic was applied to autosomes for males and all chromosomes for "females".

Point 4:
The analyses in the last section of the results (Lines 340-360) are highly expected and the analyses in Figure 5 seem to be too rough and not very informative. Comparing the center and the arm regions of the chromosomes are very rough categorization regarding the epigenetic state surrounding the genes. It is generally expected that regulatory elements evolve independently from the local divergence of neutral sites. Therefore, I feel that these analyses can be omitted or moved to supplementary materials.

Response:

>>> Following the reviewer suggestion, we have moved most of the original Figure 7 to the supplement, retaining the plot demonstrating that correlations between cis regulatory divergence and coding sequence divergence are positive but weak.

Minor comments:

Line 94-98: Does “this work” refer to the submitted manuscript? The sentences seem to be at a rather odd place, although I do agree that this reported case is limited to only two species in the genus.

Response:

>>> We changed that sentence to "Overall, previous work ..." to make it clear is not our own work.

Figure 1: There was no explanation about the colors in Fig. 1B. I assumed that darker colored bars indicate significant genes. Also, what I read from Fig. 1C was that genes with higher expression in C. nigoni are enriched and those with higher expression in C. briggsae are depleted on the X chromosome. However, the sentences in lines 147-151 and 390-392 states differently (the species are opposite). Am I missing something?

Response:

>>> We now include a clearer definition of color and contents in Fig 1, as suggested.

Line 147: A brief description about the synteny of the chromosomes between the two species would be informative.

Response:

>>> We added this information in the Methods section (Ortholog identification and read abundance quantification): "The chromosomes in the *C. briggsae* and *C. nigoni* genomes are largely colinear, with only a few small inversions and translocations reported (97). Therefore, we quantified gene expression abundance for a set of 13,636 genes that we inferred to be one-to-one reciprocal orthologs between *C. briggsae* and *C. nigoni*, for which upstream regulatory regions should also be syntenic.".

Line 229-232: I did not see the pattern mentioned here to be clear from Fig. 3.

Response:

>>> We have rewritten this part of the text to be more explicit about this particular result.

Figure 4A: “interaction” and “species” column names should be opposite.

Response:

>>> Thank you for pointing this out. The figure has now been corrected accordingly.

Line 286-288: I could not read this from Fig. 4AD. Relative to which category?

Response:

>>> This is referring to the proportion of genes with C. briggsae-like or C. nigoni-like expression in F1 males. In general, genes with expression differences between species, sexes, and/or their interaction in most cases show a higher proportion of C. briggsae-like genes, regardless of pure species had higher expression in those vertical groups (first red or blue box). This whole section was completely rewritten and most of these lines were cut.

Line 312-314: Where can I find the species-by-sex interaction data that shows masculinized expression profile for C. briggsae hermaphrodites? Is this data not shown?

Response:

>>> We have prepared a reaction-norm-type figure with normalized expression showing expression tendencies across species and between sexes (Fig. 5B and Supplementary Figure S4 in the revised version). This figure includes the expression data showing masculinized expression profile for C. briggsae hermaphrodites.

Figure 5 caption: “1-Pcons” is described as “5 bp windows within 500 bp upstream of each gene”, which is not the same as what is explaned after (A) in the legend. I am not sure if “1-Pcons” is the best way to name this index.

Response:

>>> This part of the old Fig. 5 was sent to the Suppl. Mat. However, we expanded the definition in the caption of Suppl. Fig. S7 to be more precise. While 1-Pcons might not be the simplest definition of this index, we felt it was less misleading than something like "Pdiv" because a conserved window is more straightforward to define than a divergent window.

**Reviewer #2:**

Summary of major comments:
The biggest concern I have about the manuscript is the way that expression differences in X-linked genes in males are interpreted and compared to inferences made for the same genes in females. These, by necessity, must be done in different ways and it is not clear how they can be directly compared. This is also a very complicated study, with multiple different classification schemes, with different naming conventions, and relationships to terminology. The mix of terms used is sometimes hard to make sense of with respect to whether they are considered to be distinct or similar term are being used to avoid repetition. This manuscript would be improved by addressing issues of clarity, streamlining, filling in omissions, and reorganization and contextualization to make the meaning of comparisons for X-linked genes across sexes more transparent for readers. Overall, this work is a valuable contribution to this field because it makes a unique and interesting contribution to the existing body of work on hybrid sterility and inviability, expression and molecular evolution, in these species and in other nematodes.

Response:

>>> In this revised manuscript, we have updated the categorization of cis and trans-regulatory changes for transparency and clarity of meaning. In particular, we realized that it was a mistake to lump cis + trans and cis x trans into a single "enhancing" category and, therefore, we now keep both categories separate. In addition, cis and trans-regulatory changes in hybrid males are re-defined, only retaining categorizations that are unambiguous (cis-only, trans-only, and cis-trans compensatory). X-linked genes in males are also analyzed separately so that there is no confusion over false equivalency of categorizations (Fig. 3).

>>> To ease the complication and consistency between terms and categories, we have re-rendered our visualizations using color palettes that we hope are easier to distinguish and which are also color-blind friendly. Additionally, we have revised the manuscript for clarity and consistency among the terms and naming conventions.

Major Comments:

Point 1:
Patterns observed for X-linked genes in males are compared to those found in females and this appears to be interpreted as primarily due to biological differences. However, I think the two ways of classifying differences are not easy to directly compare. This creates distinct patterns that reflect, in part, differences in classification criteria (see Figure 3). How do the classifications of expression patterns for X-linked genes in males (page 28 lines 729-733) explicitly relate to the classifications for these same genes in females?

I completely understand that this is done out of necessity, but it is also tricky because losing information from the second allele (as is true for males) does not give you the same ability to separate the categories cis-only, trans-only, ‘enhancing’ or ambiguous from one another in the same way. For example, the effects of trans acting factors that are X-linked will not be separable from cis effects.  Another example is that underdominance for expression of X-linked female genes could go in the trans only category – but if I am following correctly, for X-linked genes in males, this would always go in the enhancing or compensatory category.

It seems like this might need a more contextualized and explicit treatment to ensure that it is not confusing or misinterpreted by the reader – perhaps splitting out the results involving cis, trans etc. categories for male X-linked genes and then discussing the comparisons in this context with notes on limitations.

Response:

>>> Thank you for these helpful observations, which echo the same issue raised by Reviewer #1. To reiterate: We also had concerns about how valid it would be to compare male X-linked to females and autosomes. Correspondingly, we have updated and clarified how patterns of regulatory divergence can be quantified and inferred, albeit in a more limited fashion, for X-linked genes in hybrid males from the kind of data that we present. At least three types of regulatory divergence patterns can be confidently identified, with some limitations:

- First, X-linked cis-only regulatory divergence would necessarily involve gene expression in F1 males matching expression from C. nigoni. Divergence in trans-regulatory factors would lead to deviation of this pattern, either upwards or downwards. The assumption that the paternal C. briggsae X chromosome does not contribute trans-regulatory factors is also implicit for hybrid male autosome regulatory divergence (i.e. it would be an assumption also applied to autosome genes in males). We refer to this class of genes as “cis-only*”.
- Second, X-linked genes in male hybrids with expression that matches levels observed in C. briggsae males would imply that there is no significant regulatory divergence due to cis-acting factors, with the implicit assumption that any C. nigoni trans-acting factors are recessive. This partial view likely underestimates total trans-regulatory factors affecting X-linked genes, so we have decided to mark the distinction between trans-regulatory divergence for autosomes and the X for males and females (i.e., X-linked trans-regulatory changes for males involve a subset –underestimation– of those observed for autosomes in males and for both the X and autosomes for females). We refer to this class of genes as “trans-only*”.
- Third, X-linked cis-trans compensatory changes can be identified in cases where there are no significant differences in expression between parent species despite significant deviations in expression in hybrid males. Exposure to both C. briggsae and C. nigoni trans environments would lead to such overdominant and underdominant expression among X-linked genes in hybrid males as a consequence of the evolution of compensatory factors in regulation.
- All other genes were combined into an "other" category. This logic was applied to autosomes for males and all chromosomes for "females".

Also see comment 5., supplemental tables that include expression estimates and categories would make this easier to understand.

Response:

>>> All data tables are now available through our GitHub repository ([https://github.com/santiagosnchez](https://github.com/santiagosnchez/CodonAlign)/competitive_mapping_workflow).

Point 2:
It is not clear to what degree the procedure described on pg 25 lines 631-634 eliminate map bias, especially because the references are not specific to the strains used. Since the approach is used for the allele-specific expression comparisons and not for the parental expression comparisons, this could be important. In other, similar studies, comparable procedures have not entirely eliminated map bias (see references included at the end of the review). So, it is important to know the degree to which this impacts the results – at least to make it clear that the effect is negligible. Details regarding the accuracy of the competitive mapping approach are not clear and/or appropriately cited. This should be clearly described in the manuscript. This could include a plot like that in Figure 1B for the relative expression of the alleles, results using simulated reads or other controls (although I understand that additional experiments are not feasible).   

Response:

>>> While we recognize the potential issue of mapping bias, two key factors mitigate against it in our analyses. First, we use strain-specific genome references for matching reads to each parental data set. Our transcriptomes for C. briggsae use the isogenic strain AF16, which is the same strain genotype used for the CB4 reference assembly (https://www.ebi.ac.uk/ena/browser/view/GCA_000004555.3). For C. nigoni, we use transcriptomes from strain JU1421; the reference genome comes from strain JU1422 (Yin et al. 2019). Both JU1421 and JU1422 are inbred derivatives of the isofemale strain JU1325 and therefore we expect genetic variation to be very low between them.

Second, thanks to your suggestion, we tested, improved, and validated our competitive mapping approach using simulated read data. Initial assessments and validations led to major upgrades in our code that we now incorporate into the updated analyses in this revised manuscript. These upgrades include: 1) direct and probabilistic estimates of allele-specific read counts for ambiguous reads (equally good mapping reads); previously we ignored those reads in both hybrid and parental samples; 2) our new code now outputs read counts directly instead of BAM files with best matching reads; we validated our read counting algorithm making it compatible with featureCounts (previously we were using HTseq-counts). After a second round of validation using simulated read data, we found high accuracy rates in our method for different allele-specific expression and regulatory divergence scenarios (Supplementary Figs. S11,S12). We are currently preparing a short communication to publish our method in *Bioinformatics*. All the results in the current version have been updated with the new read counts. In addition, we find substantial agreement between the results generated by our new, validated method to our previous results.

This validation step also allowed us to identify potential reproducibility and statistical inconsistency issues with the edgeR and limma approach that might introduce error. In contrast, DESeq2 behaved more consistently. Therefore, all differential expression tests in this revised manuscript were conducted using DESeq2.

Point 3:

Studies in other organisms, and with varied approaches, found that trans differences in expression and dominance coincide (e.g. Lemos et al. 2008). If I understood correctly, this is not consistently observed in this study (pg 10 lines 230-236; see also discussion pg 21 lines 538-549). I was not sure how to interpret this, because it is hard to understand mechanistically dominance for expression would occur in interactions between alleles that differ in cis, only. Is there a proposed mechanism that would result in dominance for expression that is cis-only – or is this an outcome of the way cis-only and trans-only are classified? In addition, it was not clear why trans-only effects on X-linked genes in males (pg 13 line 325-329), which are related to dominance, were classified as ‘misexpressed and misregulated’ by default.

Response:

>>> We agree that most studies associate trans-acting variation with deviations from additivity (i.e. expression dominance) due to the masking effects of dominant vs. recessive effects, and cis-acting variation resulting in more additivity (Lemos et al. 2008). However, there are several relevant differences between these studies and ours that are worth noting.

- First, Lemos et al. (2008, PNAS) is based on intra-specific variation in population of Drosophila melanogaster. Differences in mutational target size and effect size between cis and trans-acting factors can cause cis-acting changes to accumulate more slowly than trans-acting changes (Denver et al. 2005). It is possible that the cis-regulatory divergence seen between species involves many more regions than at the population level resulting in possible deviations from additivity.
- Second, some studies based on Drosophila interspecies hybrids (i.e. Wittkop et al. 2008; McManus et al. 2010) focused on females only; our data for females is largely consistent with these previous inferences for fly females.
- Third, if we look at data for trans-regulatory changes only, then the results are actually quite consistent with the expected non-additivity in both sexes (Fig. 4). If you look at additive genes alone, we do observe higher association with cis-only changes (males) or regulatory changes that have significant cis-acting changes (cis + trans; females). We agree that the inconsistency relates to genes with cis-only effects showing deviation from additivity distinctively between sexes (Fig. 4). In females, cis-regulatory changes largely associate with genes classified under non-additive expression inheritance categories, many of which are overdominant. In males, cis-regulatory changes are more associated with simple dominance or with additive expression effects.

With these results in mind, we can think of three potential mechanisms that might promote cis-dominant effects. 1) Evolution of higher or lower affinity of trans-acting factors disproportionately affecting one species. In our system, we might expect relaxed selection on male-related phenotypes and expression in C. briggsae due to their rarity in populations, potentially leading to mutational degradation of binding sites. Consequently, we might expect higher abundance of genes with cis-acting factors that associate with C. nigoni dominance; however, this is opposite to the trend that we observe. 2) Post-transcriptional regulation may cap expression in pure species and not in hybrids, leading to deviations from additivity. Merritt et al. (2008, Curr. Biol.) have shown C. elegans oogenic germline gene networks to depend strongly on 3'UTR post-transcriptional regulation, but interestingly genes showing spermatogenic expression rely primarily on upstream transcriptional regulation. It may be that sex differences in the dominant mode of regulation (transcriptional versus post-transcriptional) contributes to the sex differences in cis-dominance that we report. 3) The evolution of lineage-specific trans-factors through gene duplication and loss could potentially lead to *trans*-acting allele-specific expression. We have incorporated some of these ideas in the results and discussion sections in the manuscript.

>>> For the reviewer’s second point, only genes that show dominant expression relative to C. briggsae on the X chromosome are deemed 'misexpressed and misregulated'. Given that hybrid males have the C. nigoni X chromosome, deviations from either "conserved" regulation/expression or genes showing dominance relative to C. nigoni (cis-only regulatory divergence) would necessarily be considered as "misexpressed" because the expected expression outcome is that found in C. nigoni, and "misregulated" due to the effect of heterospecific trans-regulatory factors in hybrids. We have updated the text to make this distinction clearer.

Point 4:

I was not able to entirely follow the terminology used for classifying patterns of expression. For example, on pg 12 lines 295-299, it sounds like overdominance would not be considered misexpression. However, on pg 13 line 320-321 the term is used to encompass overdominance or underdominance, as I expected. In general, making the language more consistent and clarifying how the terms misregulation and misexpression are defined in this manuscript would be helpful. Specifically, if these terms are different from just transgressive expression – it should be clear why/how.

Response:

>>> Thank you for pointing out this ambiguity. We have aimed to use consistent and clear language throughout our revised manuscript. In this specific example involving pg 12 and 13, we now restrict our wording to use “misexpression” to avoid confusion. We agree with the reviewer that both overdominance and underdominance are considered a signature of misexpression, as we indicate on p 12. We have revised the manuscript to make terms like misexpression, misregulation, and transgressive expression clearer.

Point 5:

I expect this is in the process of being completed – but submissions to GEO and SRA are incomplete. In addition, I was surprised that there were no supplementary tables with summary statistics etc.? At a minimum this would include expression estimates, output of statistical analyses, information that links genes to the various category assignments, including M0-M12, and the designation of spermatogenesis gene.

Response:

>>> Raw sequencing data is now on NCBI and we have made available raw gene-wise read counts files, workflow scripts that include data processing and read-mapping, R scripts with differential expression analyses, statistical analyses, and plotting functions through a GitHub repository. In addition, we are providing specific links after each relevant section in the Methods to refer to summary tables and raw data.

We have also edited the Data Availability statement to reflect those changes:

" Raw sequencing data can be found on the Short Read Archive under accessions SRR12532505- SRR12532522 and BioProject PRJNA659254. Raw gene counts, workflow scripts, and main data tables can be found on GitHub under the following repository: [https://github.com/santiagosnchez](https://github.com/santiagosnchez/CodonAlign)/competitive_mapping_workflow."

Minor comments:

1.      Despite the difference in the way things are classified for X-linked genes, the difference in dominance relationships for X-linked genes as compared to those on autosomes (pg 16 lines 390-393; also see pg 17 lines 421-423) was especially intriguing with respect to the possibility of divergence of genes and pathways involved in dosage compensation. I think a discussion of these results should include this possibility in some way, at least to explain that these mechanisms aren’t expected to impact adult expression (although if it is misexpression or different from C. elegans?).

>>> Thank you for suggesting this idea. Given the strong pattern of down regulation (i.e. underdominance) that we observe in F1 hybrid females, we thought that divergent dosage compensation (DC) mechanisms in C. briggsae and C. nigoni might potentially act to excessively supress expression. However, research from Barbara Meyer’s lab shows that DC complex (DCC) motifs are conserved between these species, indicating conserved mechanism of DC (https://escholarship.org/uc/item/2w2568f1). DC in Caenorhabditis acts in females and hermaphrodites to reduce expression by half for both X chromosomes (http://dx.doi.org/10.1895/wormbook.1.8.1). We compared the overall expression magnitude for autosomes and the X in females (Suppl. Fig. S1), and observed no general signature of disruption of dosage compensation leading to exceptionally low levels of expression in F1 hybrid females. We added a section to the discussion on dosage compensation and we also refer to Suppl. Fig. S1.

2.      There is some awkward phrasing throughout. Some of the longer sentences are grant proposal like. Breaking them up would improve readability.

>>> We have revised the manuscript to improve the readability, with special attention to long sentences.

3.      There seem to be a lot of places where reviews are cited, but original work is omitted from the citations. For one example see pg 18, line 463. It would be natural to cite Palopoli et al. 2015 here.

>>> Thank you for pointing out this reference. We have added it to the current version. We have also revised other citations to include additional original work.

4.      This is a complex paper with a lot of different components and is relatively long. This is not necessarily a bad thing, but it can decrease the ability of readers to follow all the ‘threads’ and therefore reduce overall impact of the paper. If length and complexity need to be reduced, the sections related to comparisons across chromosomal regions (center, arm – but not ends?) do not seem to underlie or tie into a major conclusion and so could be reduced, removed or moved to the supplement.

>>> Thank you for the recommendation. We agree that is somewhat complex and tries to follow many different subject threads. We have moved most of this analysis to the supplement, as suggested. We felt it valuable to retain one plot related to molecular evolution to demonstrate that the positive association between coding and cis-regulatory divergence is weak, not strong as might be naively intuited.

5.      Does the term ‘expression bias’ (pg 15 line 385) as it is used here mean biological differences in average expression levels or does it encompass map and other technical biases that contribute to systematic error in estimates of expression? Does the statement that there is no bias also apply to comparisons of allele-specific expression in the F1?

>>> In this case, we are referring to a biological bias in average expression differences between species. We did not mean to address biases in allele-specific expression, nor other kind of technical biases (i.e. reference map biases). We therefore adjusted the text as:

"While in some systems, such as fruit files and plants, expression differences between species are often biased towards one species".

We have also updated Fig. 1 to include a histogram for allele-specific expression, which should address potential concerns about map-biases.

6.      ‘Enhancing effects’ was confusing terminology for me (Pg 28 719-721): “Genes with either significant 720 synergistic (cis + trans) or antagonistic (cis x trans) cis-trans effects were grouped together as 721 representing changes with enhancing cis-trans effects. “

The cis x trans category would be alleles that change direction between the parental comparison and the hybrid comparison, following from McManus et al. 2010. I could not wrap my mind around how this was enhancing – this language seemed like it would refer to the direction of cis and trans effects being the same, but here I think we would infer that they are opposing in direction (but not exactly equal as they would be in the compensatory case). 

>>> Thank you for pointing this out. We've now realized that this grouping (cis x trans and cis + trans as "enhancing") is, in fact, erroneous. Cis + trans is the only group that should include enhancing effects. Cis x trans is a special case of compensatory cis-trans effects where there is an offshoot of expression caused by the compensating factors. We have now updated the regulatory divergence groups leaving the original 7 categories.

7.      Pg 4, lines 81-84. True and Haag 2001 should be cited here (it is in the references already, just cited later).

>>> Citation added.

Palopoli MF, Peden C, Woo C, Akiha K, Ary M, Cruze L, Anderson JL, Phillips PC (2015) Natural and experimental evolution of sexual conflict within Caenorhabditis nematodes. BMC evolutionary biology, 15:93. <https://doi.org/10.1186/s12862-015-0377-2>

Degner JF, Marioni JC, Pai AA, Pickrell JK, Nkadori E, Gilad Y, Pritchard JK (2009) Effect of read-mapping biases on detecting allele-specific expression from RNA-sequencing data. Bioinformatics , 25(24):3207–3212. <https://doi.org/10.1093/bioinformatics/btp579>

Graze RM, Novelo LL, Amin V, Fear JM, Casella G, Nuzhdin SV, McIntyre LM (2012) Allelic imbalance in Drosophila hybrid heads: exons, isoforms, and evolution. Molecular biology and evolution, 29(6):1521–1532. <https://doi.org/10.1093/molbev/msr318>

Pandey RV, Franssen SU, Futschik A, Schlötterer C (2013) Allelic imbalance metre (Allim), a new tool for measuring allele-specific gene expression with RNA-seq data. Molecular ecology resources, 13(4):740–745. <https://doi.org/10.1111/1755-0998.12110>

Lemos B, Araripe LO, Fontanillas P, Hartl DL (2008) Dominance and the evolutionary accumulation of cis- and trans-effects on gene expression. Proceedings of the National Academy of Sciences of the United States of America, 105(38):14471–14476. <https://doi.org/10.1073/pnas.0805160105>

Rodriguez MA, Vermaak D, Bayes JJ, Malik HS (2007) Species-specific positive selection of the male-specific lethal complex that participates in dosage compensation in Drosophila. Proceedings of the National Academy of Sciences of the United States of America, 104(39):15412–15417. <https://doi.org/10.1073/pnas.0707445104>

>>> Thank you for pointing out these useful references.

**Reviewer #3:**

Point 1:
My first major suggestion is that the study should explicitly consider the anatomy and morphology of both species of worms and their hybrids when interpreting their results.  I think a good treatment of the issues that can arise when assaying gene expression from whole animals can be found in Ranz et al 2004, doi:10.1101/gr.2019804.  After some digging through the literature, it appears that some of my questions listed below may be answered by Woodruff 2010 (reference #46) but the authors may also have data to more directly address these issues.

Response:

>>> From our own work, and also indicated in Woodruff et al. (2010), hybrid females are fertile and superficially normal in morphology and anatomy (albeit with some developmental delay in gonad maturity) whereas hybrid males are sterile with defective gonad development and tend to be smaller overall than pure species males. We now discuss the potential influence of gonad:soma allometry, though we were unable identify a particular part of the Ranz et al. (2004) methodology that we could adopt with our existing data. They "controlled" for allometric expression by generating additional tissue-specific (head) microarray data sets, but still interpreted their whole-body results in the same way that we do. Given our current constraints, we aim to be forthright with readers that allometric relationships with respect to hybrid morphology was not explicitly controlled for and that this allometric relationship might impact the interpretation of some results, which we now raise in the Discussion.

Point 2:
First, inferences regarding gene expression evolution between the females of the two species may be complicated by the fact that C. briggsae is androdioecious.  While this is noted in the manuscript, it didn’t seem to me that this fact was ever explicitly integrated into the analyses; instead in the methods it was stated that the only morphological difference between briggsae hermaphrodites and nigoni females is a small population of spermatogenic cells in the former.  However, treating these hermaphrodites as females is undermined by the observation of masculinization of briggsae hermaphrodites relative to nigoni females (Fig. 1A, line 163-164). I think some effort should be made to determine the extent to which the spermatogenic cells contribute to differences in gene expression profiles derived from whole animals.  Possible approaches include looking at the expression of male-biased or spermatogenesis genes and estimating the extent to which these might contribute to
differences in expression between briggsae hermaphrodites and nigoni females, and/or to compare expression differences between briggsae and nigoni females to expression differences between wild-type and fog genotypes (or other feminizing mutations) in C. elegans. 

Response:

>>> Thank you for bringing up this issue, which also was raised by another reviewer. To reiterate: As suggested, we have added information and explanation about the sexual system to the introduction and the methods sections, including the hybrids (they are females, not hermaphrodites). Hermaphrodites and females share the same somatic phenotype, but hermaphrodites have a gonad that transiently makes sperm cells. To improve the biological appropriateness of comparisons of the sexes, we now incorporate analysis of differential expression between C. briggsae hermaphrodites and C. briggsae she-1 mutants, a mutation which precludes spermatogenesis in hermaphrodite gonads, making them "pseudo-females" (data from Thomas et al. 2012). With this additional analysis, we identify a set of about 1,000 genes that are male-biased and also upregulated in hermaphrodites (Fig. 6). These genes are enriched for orthologs that are upregulated during spermatogenesis in C. elegans (Ma et al. 2014) and for a cluster of male-biased genes that are upregulated in C. briggsae hermaphrodites, having a species-by-sex interaction (B-M-I; Figs. 5 and 6). Thus, we interpret this group of ~1,000 genes as representing hermaphrodite-expressed genes associated with male function (i.e. spermatogenesis). In our efforts to mitigate potential biases due to differences in mating systems, we have excluded this set of genes from all analyses where C. briggsae hermaphrodites and C. nigoni females were compared.

Point 3:
I think it is important to understand the potential contribution of differences in anatomy/morphology between these species to differences in gene expression, as this explanation is rather distinct from the more direct gene regulatory paradigm frame employed in this manuscript.  It may also be significant for interpreting the observation of an excess of species differences in gene expression in females relative to males. 

Response:

>>> The morphology of Caenorhabditis species is highly conserved even between very distantly related species, making species difficult to distinguish morphologically, even for specialists (Barriere and Felix 2014 Wormbook). The total number of somatic cells in adults is about 1,000 in C. elegans, the cell fates most of which are established in embryogenesis which is nearly indistinguishable among species despite reproductive isolation (Memar et al. 2018 DevBio); animals have an additional 1000-2000 germline cells depending on the sex. Body size is very similar for C. briggsae and C. nigoni (1.1mm to 1.2mm long), and body size differences within and between species reflects cell size differences not different cell numbers (Woodruff et al. 2018 EvolLett). Morphologically, females and hermaphrodites share a highly similar somatic phenotype. An indication of an effect of allometric scaling between species would be seen as bias in expression divergence towards the species of the larger body size (i.e. Stephen and Mank 2016 Molecular Ecology), but we do not observe this in our data (Fig. 1A). In fact, the number of differentially expressed genes upregulated in each species seems to be symmetric. Also, there haven't been reports that F1 hybrid "females" are anatomically different, other than they have delayed germline development (Woodruff et al. 2010). The only anatomical issue that we can identify is that F1 hybrid males are typically smaller and have malformed gonads compared to males in pure species. In spite of this, our data and analyses show that males have more genes with "conserved" expression and regulation (categorized as "no change" in expression inheritance or "conserved" in regulatory divergence groups) than "females". We now comment on some of these issues in the Discussion.

Point 4:
A related question involves the morphology of the F1 “female” hybrids.  Are F1 offspring from this cross females or hermaphrodites? (Woodruff 2010 suggests mostly females)  Again, the morphology of these F1 offspring may be relevant for interpreting whether there are anatomical explanations for differences in gene expression between either parental species and F1 females in addition to the effects of evolved and perturbed gene regulatory networks.

Response:

>>> As you mention, Woodruff et al. (2010) affirmed that XX F1s are females based on the exceptionality of laid eggs and spermatocytes when left in isolation. Woodruff et al. (2010) were not able to detect molecular markers for spermatogenesis in F1 females (FOG-3, MSP). Interestingly, our data shows that hybrid females express low levels of fog-3, which is an activator of germline development, and is dominantly expressed relative to C. nigoni (i.e. normal female germline development). In contrast, in F1 males fog-3 is misexpressed (underdominant), which might contribute to the observed phenotype of underdeveloped gonads.

>>> Anatomically, XX F1s do not show major morphological or anatomical abnormalities compared to hermaphrodites and females. There is some speculation as to why F1 female hybrids are not hermaphrodites. Our results suggest that part of the female *C. nigoni* *trans*-environment acts dominantly upon spermatogenesis gene networks potentially supressing spermatogenesis (Fig. 6; pg. 17-18, lines: 432-458).

Point 5:
Beyond the question of female vs. hermaphrodite, the mating systems of these species might also give rise to anatomical differences that could influence whole-animal gene expression profiles.  These ideas are thoroughly considered in Cutter, Morran, and Phillips 2019 in Genetics, and I think it would be useful to apply them in this context.  This seems particularly salient given some of the unbalanced patterns of up/down-regulation, such as the large excess of female-biased genes upregulated in briggsae hermaphrodites relative to nigoni females, and potentially the excess of male-biased genes upregulated in nigoni relative to briggsae males.  Do males of these species have different allometric relationships between soma and germline as a consequence of different intensities of sexual selection?

>>> We know of no studies that explicitly analyze soma:gonad allometry across species of Caenorhabditis, and agree that it is an interesting open question. Male body width may be a proxy for gonad size (Vielle et al. 2016 Evolution), although this has not been empirically validated. In our ad hoc calculation of length:width ratio for males of C. briggsae and C. nigoni (data from Vielle et al. 2016), we find very similar values (18.7 vs. 19.1; across 27 species this ratio varies from 15.0 to 22.0). Males of C. briggsae and C. nigoni do differ in sperm cell size. However, it is not clear whether they differ in gonad tissue investment despite the prediction that C. briggsae males would invest less, given relaxed male-male and male-hermaphrodite sexual selection in this species. Given this prediction, we have added further discussion of the issue in light of our findings in this revised manuscript.

Point 6:
Finally, this same consideration should be extended to the mechanism of F1 hybrid male sterility.  Do these males produce non-functioning sperm?  Do they lack a germline entirely?  Woodruff 2010 reports that F1 males have underdeveloped gonads and no mature germ cells … if this is true for the animals used here for RNA-seq, then the large excess of male-biased genes in F1 hybrids relative to the parental species is really surprising and begs further investigation, and at a minimum more extensive discussion in the paper.

>>> As you point out, F1 males have deformed gonads and immature and scant spermatocytes. F1 males themselves also are small. The short answer is that we do not know what specific regulatory networks are being disrupted in hybrid males that cause the paucity in spermatocyte development. We hoped that our data would give us a clearer idea, but we realize that other kinds of studies and systems are needed to be able to answer those questions.

- First, new AS count data and analyses show a smaller gap between male-biased and female-biased genes, as well as overall sex-biased genes, than we previously reported.
- Second, our data suggests that the large excess of male-biased genes stems from high upregulation in hybrid males relative to females particularly in Chromosomes V and X (Fig. 1D). Many of the male-biased genes, such as M1 and M7 in Supplementary Fig. S2, sex-biased expression is actually not as strong between species as it is between hybrids, potentially causing an excess of male-biased genes. Similarly, for male-biased genes with expression divergence, such as M8 in Supplementary Fig. S2, there seems to be a sex-specific dominance effect, which artificially generates more male-biased expression in F1 hybrids. All those co-expression clusters (M1, M7, M8) are enriched in the X and partly in Chromosome V.

>>> We have added some of these points to the results and discussion sections.

Point 7:
My second major recommendation is that the analyses of the X chromosome in males needs to be revised.  The hemizygosity of the X chromosome in males obviously presents challenges for the inference of cis vs. trans regulation via allele-specific expression.  To deal with this, the manuscript appears to assume that in an F1 hybrid, all autosomal trans-regulatory loci regulating X-linked genes have dominant briggsae alleles, so that nigoni-like F1 expression is due entirely to cis-acting variation and briggsae-like F1 expression is due entirely to trans-acting variation.  No justification for this assumption is presented, and it seems unrealistic to say the least.  I *think* the only way to make X-linked cis vs trans inferences in males is to compare expression in reciprocal F1 hybrids, which seems impossible (?) with these two species. 

>>> Thank you for making this point, which also was raised by other reviewers. We have substantially revised our treatment of expression divergence on the X chromosome for hybrid males. The data that we have in hand indeed presents a challenge to infer male-X regulatory divergence in a comprehensive way. However, we find that some information regarding male-X regulatory divergence can be deduced using similar logic to the one applied to McManus et al. (2010), Wittkopp et al. (2004), and Wayne et al. (2004). This last reference, in fact, uses very similar logic to the one we apply, which is explained in detail in Suppl. Fig. S14 . To reiterate from our response to a reviewer comment above:

- First, X-linked cis-only regulatory divergence would necessarily involve gene expression in F1 males matching expression from C. nigoni. Divergence in trans-regulatory factors would lead to deviation of this pattern, either upwards or downwards. The assumption that the paternal C. briggsae X chromosome does not contribute trans-regulatory factors is also implicit for hybrid male autosome regulatory divergence (i.e. it would be an assumption also applied to autosome genes in males). We refer to these genes as “cis-only*”.
- Second, X-linked genes in male hybrids with expression that matches levels observed in C. briggsae males would imply that there is no significant regulatory divergence due to cis-acting factors, with the implicit assumption that any C. nigoni trans-acting factors are recessive. This partial view likely underestimates total trans-regulatory factors affecting X-linked genes, so we have decided to mark the distinction between trans-regulatory divergence for autosomes and the X for males and females (i.e., X-linked trans-regulatory changes for males involve a subset –underestimation– of those observed for autosomes in males and for both the X and autosomes for females). We refer to these genes as “trans-only*”.
- Third, X-linked “cis-trans compensatory” changes can be identified in cases where there are no significant differences in expression between parent species despite significant deviations in expression in hybrid males. Exposure to both C. briggsae and C. nigoni trans environments would lead to such overdominant and underdominant expression among X-linked genes in hybrid males as a consequence of the evolution of compensatory factors in regulation.
- All other genes were combined into an "other" category. This logic was applied to autosomes for males and all chromosomes for "females".

Point 8:
The results regarding patterns of gene expression inheritance in females was pretty striking, and made me wonder if differences in or disruptions to dosage compensation could be responsible?  For example, if F1 hybrid females have over-aggressive dosage compensation, could that simultaneously lead to X-chromosome wide downregulation (underdominance) and autosome-wide upregulation (overdominance) through disrupted X-autosome chromatin balance?

>>> Thank you for raising this issue, which we have looked into further given the ideas you suggested. Given the strong pattern of down regulation (i.e. underdominance) that we observe in F1 hybrid females, we thought that divergent dosage compensation (DC) mechanisms in C. briggsae and C. nigoni might potentially act to excessively supress expression. However, research from Barbara Meyer’s lab shows that DC complex (DCC) motifs are conserved between these species, indicating conserved mechanism of DC (https://escholarship.org/uc/item/2w2568f1). DC in Caenorhabditis acts in females and hermaphrodites to reduce expression by half for both X chromosomes (http://dx.doi.org/10.1895/wormbook.1.8.1). We compared the overall expression magnitude for autosomes and the X in females (Suppl. Fig. S1), and observed no general signature of disruption of dosage compensation leading to exceptionally low levels of expression in F1 hybrid females. We added a section to the results on dosage compensation and we also refer to Suppl. Fig. S1.

Point 9:
The strong excess of sex-biased genes in F1 compared to pure species result shown in Fig. 1D is not even referenced in the results and is only briefly mentioned in the discussion.  I would like to learn more about this pattern – are these genes that show mild and non-significant sex-bias in pure species, but stronger and significant sex-bias in hybrids? Are these genes that are sex-biased in one species but not the other?  And to what extent is this pattern driven by the X chromosome, specifically its downregulation in F1 females?  Does this pattern hold when considering just the autosomes?

>>> Yes, we agree this is indeed interesting. Fig. 1D gives you a hint of the distribution of sex-biased genes across chromosomes. As you suggest, Chromosomes V and X are disproportionally responsible for the overall high number of male-biased genes in F1s. In contrast, Chromosomes I, II, III, and IV are responsible for the overabundance of female-biased genes in F1s. So, this pattern would not hold if looking at autosomes and the X separately (Suppl. Fig. S2). This pattern, seems to be linked to the number of overdominant genes on these chromosomes for each sex (Fig. 2C) (i.e. a high number of X-linked underdominant genes for F1 females and a high number X-linked overdominant genes for F1 males). A large fraction of the male-biased genes in F1s are actually also male-biased genes in parent species (2,132 genes, 37%), followed by genes that are male-biased only in F1; the same genes being sex-neutral in parent species (1,331 genes, 23%). We have added more of this kind of information in the results section.

Point 10:

The results presented in lines 191-201 should be modified.  The excess of genes with briggsae dominant phenotypes in females seems to be driven almost entirely by the X chromosome, but it looks to me in Figure 2C that X-linked genes in males are equally likely to show briggsae or nigoni-like phenotypes, while the autosomes show an excess of biggsae-like phenotypes.  I think this could be clarified if the calculations of the ratio of genes with briggsae or nigoni expression phenotypes were done for each sex and for the X and autosomes separately. 

>>> Thank you for the suggestions. We have modified this section of the text to be more clear about differences in simple dominance in expression across autosomes and the X, as well as males and females:

" F_1_ hybrids of both sexes had a relatively low percentage of genes with additive expression (8% or 691 genes in females; 8% or 946 genes in males) (**Fig. 2C**). By contrast, approximately 20-30% of genes expressed by each sex showed simple dominance, either matching *C. briggsae* or *C. nigoni* expression (23% or 2,869 genes in females; 30% or 3,698 genes in males). Hybrids of both sexes consistently showed a higher number of genes with expression dominance matching *C. briggsae* (15% or 1,876 genes in females and 18% or 2,228 genes in males), compared to expression dominance matching *C. nigoni* (**Fig. 2C**; 8% or 993 genes in females and 12% or 1,470 in males). Female hybrids tended to have a slightly higher ratio of *C. briggase* vs *C. nigoni* dominant expression (mean ratio = 1.75) compared to males (mean ratio = 1.14) across autosomes (**Fig. 2C,D**). Strikingly, that difference was more pronounced in the X-Chromosome with females having over 1-fold *C. briggsae* dominant genes (F_1_ female X ratio = 2.66 vs F_1_ male X ratio = 1.13)."

Point 11:
What is the rationale for combining the categories referred to by McManus et al as “cis + trans” and “cis x trans” into a single category called “enhancing”?  At the very least, the use of the term “enhancing” is a bit misleading, since this category includes both genes where the cis and trans components promote expression of the same allele and genes where the cis and trans components promote expression of different alleles. 

>>> Our original grouping "cis + trans" and "cis x trans" into a single category was, in retrospect, inappropriate and was done, initially, to be able to compare patterns between X and autosomes in males and females. In the revised manuscript, we keep the original 7 categories used by McManus et al., with the distinction that male-X regulation is compared separately using different groupings.

>>> We like your idea of incorporating data from F1 males of the reciprocal cross. We do believe it is a promising way to generate semi-equivalent regulatory divergence categories for males and females across autosomes and the X chromosome. Unfortunately, the reciprocal cross of this species pair yields almost complete inviability of F1 males.

Point 12:
In the paragraph starting on line 290, how is it possible to disentangle regulatory divergence from hybrid dysregulation?  That is, do male-biased genes more frequently show underdominant expression phenotypes and cis-trans changes because they are less robust, or because of more evolutionary change in those networks?

>>> This is a good question; and we hope to have addressed it in more detail in the manuscript. We agree that it is hard to disentangle, as they can very well be two sides of the same coin. We suggest that a crucial underlying question is: are divergent regulatory networks more likely to be fragile networks?

>>> We have edited this paragraph to make the discussion broader about this topic.

Point 13:
I found much of the discussion, such as the section on sexual conflict (lines 451-471) quite speculative and the connections between the data presented in the manuscript and the inferences drawn from the data are often tenuous.  I think the discussion in general would benefit from narrower interpretation of the results.

>>> We have revised the discussion to narrow down and focus on tangible interpretations of our results, including the section on sexual conflict.

Minor comments

Lines 18-19: I found the juxtaposition of stabilizing and directional selection in the abstract confusing

>>> We restructured the sentence as: "Divergence in *cis*- and *trans*-acting regulatory controls can be hastened by directional selection with adaptation, sexual selection, and inter-sexual conflict, in addition to evolving unnoticed under stabilizing selection".

Line 100: what is an “outward” phenotype?

>>> As opposed to molecular phenotypes like gene expression, we use this term to refer to morphometric organismal phenotypes. We have reworded this sentence to avoid confusion.

Line 105: reference #26 did not assay gene expression in interspecific hybrids

>>> We have removed this inappropriate reference to that point.

Line 121: the introduction of the biological species concept seems odd here

>>> We have completely rewritten that section of the introduction.

Line 142: I think it would be helpful to state here that the F1 hybrids are derived from nigoni females x briggsae males, rather than requiring the reader to go to the methods for this info

>>> We have completely rewritten the last paragraph of the introduction. The new intro includes more details about F1 hybrids.

Line 205: I might substitute the phrase “patterns of inheritance” for “heritability” here and throughout.  It is more clunky, but heritability makes me think this refers the proportion of phenotypic variation due to genetics.

>>> We now refer to this term as "expression inheritance" consistently across the text.

Line 210: In F1 males there is a significant enrichment of overdominant gene expression on both chromosome V and the X.  How does this fit into the narrative of “distinct hybrid expression dynamics between autosomal and X-linked genes”?

>>> Expression dynamics that are a result of bidirectional negative interactions between autosomes (i.e., Chromosome V) and the X-chromosome can lead to extensive misexpression in those chromosomes affected by those interactions. Also, expression dynamics can involve differences in other types of regulation. For instance, genes with underdominant expression in hybrid males are enriched in the X and depleted in Chromosome V. Although this comparison pertains hybrid females, Fig. 4 shows, to a large extent, consistent regulatory and expression between across autosomes and are clearly distinct from the X-chromosome.

 Line 219: The statement that “We found most expression divergence between species results from cis-only, trans-only, and enhancing cis-trans regulatory divergence” seems like an strange point to make, given that those are 3 out of the 4 categories of divergence tabulated.  Perhaps the argument should be that less compensatory divergence was observed than expected, although I am not sure what the null expectation for the amount of compensatory divergence should be.

>>> We have restructured that section of the manuscript to make out point clearer.

Line 277: I don’t understand how these results “highlight how stabilizing selection can act differently on sex-specific transcript abundance”

>>> We reworded this part of the text to improve clarity.

Line 336: What evidence is there that any of the gene expression changes identified in this study are associated with hybrid male sterility?

>>> Yes, we don't have direct evidence (i.e. mutant assays). We rewrote this section to toned-down our arguments and reflect the limits of our analyses.

Line 379: Previous studies have assumed a 60-day generation time for wild Caenorhabditis nematodes (e.g. Cutter et al 2006, Genetics), why were generation times estimated as 36 days here?

>>> As for Drosophila, a challenge with this system is knowing the generation time in the wild, making such calculations largely dependent on educated guess. 60 days is considered a conservative generation time allowing time spent overwintering and/or as dauer larve in the wild, which can persist for months, with other calculations using 14-36 days (Cutter 2008 Mol. Biol. Evol., Thomas et al. 2015 Genome Res.). Both of these species are found most commonly in tropical and subtropical regions. We opted to follow an intermediate generation time in our calculations here.

Line 387: The discussion of demographic effects and regulatory evolution seems to be making assumptions about selection, drift, and the direction of regulatory changes (increased or decreased expression) that are not clearly stated, and likely unsupported (I don't know of any theory or data suggesting up- or down-regulation is more likely to be beneficial, deleterious, or neutral).

>>> We have rewritten that section of the discussion.

Line 434: Reference #28 does not report an excess of X-linked misexpression in sterile hybrid males.  In contrast to some of the arguments presented in reference #68, the evidence that X-linked gene misexpression contributes to hybrid male sterility in flies is basically nonexistent (in my opinion).

>>> Our statement does not argue in favor of an "excess" of X-linked misexpression. It simply references cases where X-linked misexpression has been associated with hybrid male sterility. In that particular reference (Gomes and Civetta 2015), they mention that at least one misexpressed X-linked gene is found within a QTL associated with hybrid sterility, although they do mention that many more (14) were associated with Chromosome III. In any case, we have removed the citation from the list.

--------------------
