# Supplementary material for: Widespread misregulation of inter-species hybrid transcriptomes due to sex-specific and sex-chromosome regulatory evolution
Source: PLoS Genet. 2021 Mar 5;17(3):e1009409. doi: 10.1371/journal.pgen.1009409 (PMC7968742; doi:10.1371/journal.pgen.1009409)
Supplement: Attachment — Submitted filename: reply_to_reviewers.docx [file pgen.1009409.s015.docx]

Dear Editors,

Thank you again for taking our resubmission into consideration. We appreciate the time and effort taken by the reviewers to read and assess our manuscript. We have now updated our manuscript based on the comments below and have also answers to the concerns of the reviewers in detail. In particular, we have clarified, either by a direct reply and/or on the text, two main issues raised by Reviewer #3 regarding 1) cis-only divergence on the X chromosome, 2) distinguishing between two competing hypothesis about "fragile" male genes.

We hope that with these changes the manuscript would be acceptable for publication in PLoS Genetics.

Kind regards,

Santiago Sanchez-Ramirez & Asher Cutter

**Editors**:

Dear Dr Sanchez-Ramirez,

Thank you very much for submitting your Research Article entitled 'Widespread misregulation of inter-species hybrid transcriptomes due to sex-specific and sex-chromosome regulatory evolution' to PLOS Genetics.

The manuscript was fully evaluated at the editorial level and by independent peer reviewers. The reviewers appreciated the attention to an important topic but identified some concerns that we ask you address in a revised manuscript

We therefore ask you to modify the manuscript according to the review recommendations. Your revisions should address the specific points made by each reviewer.

>>> The manuscript has now been revised accordingly.

In addition we ask that you:
1) Provide a detailed list of your responses to the review comments and a description of the changes you have made in the manuscript.

>>> We made a list summarizing changes made to the manuscript. Please see below our responses in detail.

2) Upload a Striking Image with a corresponding caption to accompany your manuscript if one is available (either a new image or an existing one from within your manuscript). If this image is judged to be suitable, it may be featured on our website. Images should ideally be high resolution, eye-catching, single panel square images. For examples, please browse our [archive](http://www.plosgenetics.org/article/browse/volume). If your image is from someone other than yourself, please ensure that the artist has read and agreed to the terms and conditions of the Creative Commons Attribution License. Note: we cannot publish copyrighted images.

>>> A Striking Image has been included in this submission.

We hope to receive your revised manuscript within the next 30 days. If you anticipate any delay in its return, we would ask you to let us know the expected resubmission date by email to [plosgenetics@plos.org](mailto:plosgenetics@plos.org).

If present, accompanying reviewer attachments should be included with this email; please notify the journal office if any appear to be missing. They will also be available for download from the link below. You can use this link to log into the system when you are ready to submit a revised version, having first consulted our [Submission Checklist](http://journals.plos.org/plosgenetics/s/submit-now#loc-submission-checklist).

While revising your submission, please upload your figure files to the [Preflight Analysis and Conversion Engine](http://pace.apexcovantage.com/) (PACE) digital diagnostic tool. PACE helps ensure that figures meet PLOS requirements. To use PACE, you must first register as a user. Then, login and navigate to the UPLOAD tab, where you will find detailed instructions on how to use the tool. If you encounter any issues or have any questions when using PACE, please email us at [figures@plos.org](mailto:figures@plos.org).

>>> Our images have been assessed and processed by PACE.

Please be aware that our [data availability policy](http://journals.plos.org/plosgenetics/s/data-availability) requires that all numerical data underlying graphs or summary statistics are included with the submission, and you will need to provide this upon resubmission if not already present. In addition, we do not permit the inclusion of phrases such as "data not shown" or "unpublished results" in manuscripts. All points should be backed up by data provided with the submission.

>>> All our data and scripts are publicly available either through NCBI or GitHub.

PLOS has incorporated [Similarity Check](http://www.crossref.org/crosscheck.html), powered by iThenticate, into its journal-wide submission system in order to screen submitted content for originality before publication. Each PLOS journal undertakes screening on a proportion of submitted articles. You will be contacted if needed following the screening process.

To resubmit, you will need to go to the link below and 'Revise Submission' in the 'Submissions Needing Revision' folder.


Please let us know if you have any questions while making these revisions.

Yours sincerely,

Alex Buerkle
Associate Editor
PLOS Genetics

Bret Payseur
Section Editor: Evolution
PLOS Genetics

This revised manuscript has been reviewed by three referees, who concur that the revisions have substantially improved the manuscript and that only minor points remain to be addressed. I encourage the authors to consider these additional suggestions for improvement of the manuscript.

**Reviewer's Responses to Questions**

**Reviewer #1**:

I think that the authors have addressed most of the issues I have raised previously. There are still several points that need to be considered before publication.

1. The updated and clarified treatment of the regulatory divergence categorization of the X-chromosome in hybrid males (described in lines 315-334, 939-963, and Figure S14) is now reasonable. However, I found some inconsistent statements regarding the trans-only category. In lines 326-328, the authors mention that they found only a non-significant trend in both sexes toward enrichment of trans-only regulatory divergence affecting X-linked genes, but in the Figure 3 legend, it says that the X-chromosome is enriched for trans-only changes in both sexes despite that Figure 3A does not show significant enrichment of trans-only category in either sex.

>>> Thank you for pointing out this inconsistency. We have now updated the legend of Figure 3 by indicating that only cis-trans compensatory changes are significant in males, and that trans-only* changes are nominally enriched but non-significant in both sexes (log odds ratio > 0).

Also, lines 695-698 (in Discussion) and line 746 (in Conclusion) refer to the trans-only effect being significant. In addition, there is no description about how "conserved" category indicated in Figure 3 was scored.

>>> We revised those lines in the Discussion to avoid misleading wording. The changes the the Discussion were:

"**Although enrichments on the X were non-significant**, we find **other signs** that *trans*-acting factors contribute to misexpression in both sexes (**Fig. 3, 4**). In hybrid females, *trans*-acting factors largely drive the expression of X-linked genes with *C. briggsae* dominant and underdominant expression, unlike autosomes (except Chromosome V) (**Fig. 4**). In hybrid males, our inference that all X-linked genes with *C. briggsae* dominant expression arise from *trans*-only* effects implicat**ing** recessive *C. nigoni* *trans* regulators (**Supplementary Fig. S14**), **and** which, at minimum, should be an underestimation of all *trans*-acting regulatory divergence affecting the X-chromosome, **suggests that similar *trans*-acting misregulation occurs**. These findings are consistent with previous observations, particularly in *Drosophila*, of *trans*-acting sex-specific changes causing misregulation of X-linked genes (39,88,89)."

>>> We updated the conclusion section in the following way:

"Despite the rarity of sex-biased genes on the X-chromosome, the X is home to disproportionate misexpression in both sexes, with misregulation in hybrid males largely occurring through *cis-trans* compensatory changes, **but also by *trans*-acting factors to some extent**."

>>> We don't fully agree that this line in the conclusion is misleading, as it is not only referring to significant enrichment of trans-only genes, but also to the association between *trans*-only factors and misexpression on the X in the form of underdominance in the case of hybrid females. Similarly in the case of hybrid males, all trans-only* genes are assumed to be both misregulated (C. briggsae trans factors acting on C. nigoni alleles) and misexpressed (regulatory divergence between species).

2. The numbering of the figures do not follow the order cited in the text. In line 285-293, Figure 5 is cited after Figure 2 and Figure 3 comes after Figure 4.

>>> Thank you for pointing out this oversight in number during the process of reorganization in our prior revision. We have now reordered the paragraphs under "*cis* and *trans* regulatory divergence modulates differences between sexes" to be consistent with the figure number sequence.

3. Lines 307-313 better fits in Discussion rather than in Results section.

>>> This topic is discussed in more detail in the Discussion on lines 689-698. We prefer to leave those brief points in the Results section so that the reader can right away have an idea of potential mechanisms leading to this regulatory pattern.

**Reviewer #2**:

Summary:

In this revision the authors have addressed major concerns regarding methods and interpretation with adjusted approaches and categorization. They have also done a wonderful job providing context, explanation of limitations and documentation that greatly improve the clarity and accessibility of the manuscript. I have only a couple comments, outlined below, and also provide minor suggestions for polish. The manuscript is much improved and close to being publication ready.

1. Pg. 13 lines 308-313. These are interesting mechanisms and should be included, but it seems like they would produce cis by trans effects or would not lead to dominance. Other possibilities might be transvection or differences in parent-of-origin effects?

>>> Thank you for suggesting transvection. It is indeed another option that we had not considered in sufficient depth. We have added it as a potential mechanism. In the case of the mechanisms we suggest, post-transcriptional regulation has very well the potential of causing this "cis dominance" pattern if there are epistatic interactions that limit the expression of autosomal genes to 1/2 (e.g., https://academic.oup.com/gbe/article/doi/10.1093/gbe/evp054/566807) in one or both parent species. This effect would lead to similar levels of expression for a given gene in one pure species and its corresponding allele in the hybrids. Depending on the level of expression of the downregulated gene/allele, this could lead to overdominance, which we also observe in our data (Fig. 4). We think that the third option (species-specific trans regulation) would also have a similar effect, as the expression of each allele would be controlled by distinct trans factors. This would cause significant allele-specific expression in hybrids, but perhaps some form of underdominance. We do realize that some of these are somewhat speculative, but it's all we can do in the absence of more experiments/data. The fact that we observe very little additivity suggests that other forms of regulation might be at play.

2. Chromosome V has patterns that are similar to X, relative to other autosomes. Is there any other evidence that this chromosome could be derived from an ancestral X?

>>> That's an interesting question. Previous analysis of the chromosome-scale assemblies of C. nigoni and C. briggsae show exceptional synteny across the genome with minimal inter-chromosomal translocation (Yin et al. 2018 Science), and this minimal inter-chromosomal translocation also extends to comparison of C. briggsae with the more distantly-related C. elegans (Ross et al. 2011 PLoS Genetics). This trend appears even to extend outside Caenorhabditis in comparisons to a genome in the Aunema genus (Tandonnett et al. 2019 G3). Consequently, any potential “ancient X” history of chromosome V must be very ancient.

Minor suggestions

1. I suggest ‘cryptic evolution’ instead of ‘inconspicuous evolution’.

>>> done.

2. Pg 12 line 287, Figures are out of order – currently Fig 2, 5, 4, 3.

>>> Fixed. Thanks!

3. Pg 5 line 95, adding earlier references would be nice here (e.g. Yan et al. 2002).

>>> Done. Thanks for the suggestion.

4. Figure 4 and S10 – I think the labels or categories may have a typo. They don’t match the categories given in the methods.

>>> We adjusted the description of the categories in the methods section so that it is consistent with the wording in the Figures and the rest of the manuscript.

5. Methods Pg. 34. It is a minor thing, but it might help to add that the modules are designated M1-M15 here.

>>> Done.

**Reviewer #3**:

The revised manuscript addresses most of the major reviewer concerns. The authors use gene expression data from C. briggsae her-1 mutants to control for the fact that briggsae hermaphrodites, but not nigoni or F1 females, have some spermatogenic cells. This approach and the way it informs the manuscript’s conclusions seems appropriate to me.

However, I am still not convinced that the assumptions required to infer modes of X-linked gene expression evolution in males are reasonable. The manuscript and response to reviews are clear about the assumptions being made, but does not adequately justify them, in my opinion. X-linked genes that are expressed in F1 males at levels similar to C. nigoni males are assumed to be the result of cis-regulatory evolution, but this will include (an unknown proportion of) genes regulated by C. nigoni trans-factors that are dominant in the F1. Similarly, the inferences about cis-trans compensatory evolution are confounded by under- and over-dominance just among trans-factors. The previous studies that are referenced to support their justifications don’t seem to me to be appropriate, as Witkopp 2004 and McManus 2012 used only hybrid females, and Wayne 2004 used a balanced round-robin crossing design, so at a minimum I think there needs to be a better explanation of how those studies justify the authors’ current assumptions. In short, without two alleles from which to measure ASE, or at least reciprocal males carrying different X chromosomes in the same F1 autosomal genotype, I’m not confident that much can be said about whether gene expression changes are the result of cis- or trans-divergence. I think these results, and the attendant conclusions regarding the large X-effect and hybrid male sterility, need to be revised or possibly removed.

>>> While it is true that our grouping of X-linked cis-only genes may potentially include genes regulated by trans factors that are dominantly expressed by the C. nigoni allele, we argue that this confounding factor is not exclusive of X-linked genes. Indeed, autosomal genes may equally be regulated by dominantly-expressed species-specific trans factors (we discuss 4 potential factors in the Results section leading to a "cis dominance" effect), which can violate the assumption that allelic imbalance in hybrids is directly caused cis-regulatory divergence. We decided to keep the cis-only* category, as is, given that potentially confounding factors, such as the one raised by the reviewer, are not exclusive to X-linked genes. These issues are now more clearly explained in the methods section (lines 1065-1092). Given that this categorization is novel, references to Witkopp 2004 and McManus et al. 2010 are meant to refer the reader to previous work which we have used as a foundation and inspiration for applying our logic. Indeed, Wayne et al. 2004 used a very different experimental approach in addition to being an intraspecific study. For that reason, we removed that citation. However, Fig. 2 of their 2004 paper made the leading author think that drawing information from the X chromosome might be possible. We do agree that with reciprocal crosses we would be able to validate and even draw further information. However, that is not possible with this system given that inviability reaches 95% among progeny for that cross.

line 138: is inclusion really the best word here?

>>> We reworded our only use of “inclusion” in the previous manuscript draft to: "Moreover, **extensive X-linked regions with loci causing** hybrid male sterility implicates a large-X effect".

line 179: “consistent” here seems to indicate that the pattern is the same across the different autosomes, but in the next half of the sentence it seems to indicate that the proportion of genes with higher expression of the nigoni allele vs. higher expression of the the briggsae allele are equal. If the intention is the latter, I suggest choosing a different word to make this clearer.

>>> To improve clarity, we reworded these lines as: "**The enrichment of genes where** a **given** allele gets upregulated in hybrids was largely consistent across **autosomes** of hybrid males and females (**Fig. 1B**), albeit the pattern was **slightly more extreme** for females (higher expression of *C. briggsae* allele: 3,215 or 32%; *C. nigoni* allele: 2,855 or 29%) than for males (higher expression of *C. briggsae* allele: 2,793 or 27%; *C. nigoni* allele: 2,609 or 26%)."

lines 234-236: some numbers are randomly underlined

>>> Thank you for pointing out this formatting typo. We removed the underline from the numbers.

line 347: I don’t see the connection between the observation that genes with transgressive expression in F1’s are more likely to show compensatory cis-trans interactions and “stabilizing selection can act differently on sex-specific transcript abundance”. This conclusion should be more explicitly explained. The following sentence (line 349) makes it sound like the previous sentences were reporting results for genes with sex-biased expression (?) but that is not stated above.

>>> We describe this logic on lines 82-88 of the Introduction. By definition, most transgressive expression phenotypes refer to genes that have the same overall expression levels between parents, while hybrids either have lower (underdominance) or higher levels (overdominance). The idea is that changes to both cis and trans regulators evolved in a compensatory way independently to maintain the ancestral levels of expression through stabilizing selection. These molecular changes will not be evident when simply comparing expression of parent species as there will not be any detectable expression divergence. However, in hybrids, these opposing regulatory interactions will lead to significant allele-specific expression resulting in over- or underdominance. In our analysis, we break down the results by sex. In the bottom part of the paragraph, we are actually referring to genes with no-sex biased expression, where we also find this pattern to be common. To improve clarity, we reworded those lines as: "These results highlight how stabilizing selection **can act independently on each sex to maintain sex-specific regulation and** leading to opposing *cis* and *trans* effects that **induce** dysfunction in F_1_ hybrids. In addition, we also found substantial *cis-trans* compensatory changes among genes that show no change in expression between species (**Supplementary Fig. S3**) and in those that do not show sex-biased expression (i.e., C-N-N group in **Fig. 5D**), implicating extensive developmental systems drift in regulatory processes **despite conserved profiles of expression**."

line 380: How do the authors distinguish between the hypotheses that 1) for the same number of mutational substitutions, the downstream effects on the expression of male-biased genes are greater than the effects on female-biased genes (this is my interpretation of “fragile”), vs. 2) the factors controlling the expression of male-biased genes evolve more rapidly than those controlling female-biased genes? Perhaps it would help to elaborate on what exactly they mean by “fragile”. The same question applies to the term “resilience” in the following sentences.

>>> We agree with the reviewer’s interpretation of “fragile” and also that it is useful to try to distinguish between these alternate hypotheses. We have updated the Results text in this section to reflect this point (lines 485-499). In particular, we now point out that we observed no significant difference in sequence divergence in upstream non-coding sequence overall for male-biased vs female-biased genes. We interpret this to imply that they experience similar amounts of sequence change, and therefore that it is the effects on male-biased genes that are more drastic and consistent with the notion of greater male phenotypic and gene network fragility.

line 418: The hypothesis that genes involved in spermatogenesis are depleted from the Drosophila X chromosome has been refuted (Meiklejohn & Presgraves 2012 GBE 4:895-904; Meisel et al 2012 PLoS Gen 8(10):e1003013 ) or is at least contentious.

>>> Thank you for providing these newer references. We have deleted that portion of the sentence referring to Drosophila and the older reference to demasculinized X.

lines 428-431: Are these results only considering gene expression in hermaphrodites? This is implied in Figure 6 where it appears panels D-G only include data for hermaphrodites; and makes sense considering that ASE is not possible in males for X-linked genes.

>>> No. The results from Fig. 6D-G are actually data from female F1s. Consequently, these would, in fact, use ASE data. We've updated the legend of Figure 6 to make this point clearer. This is also stated on that same paragraph line 535-534: "showed expression phenotypes in hybrids often resembling *C. nigoni* ".

line 527: I don’t see how the authors draw conclusions about the “sexually-dimorphic transcriptomes” from Figure 4, which does not break genes out by their expression differences between males and females. In general, the language throughout the manuscript should maybe be tightened and formalized a bit, to help the reader discriminate between genes differentially expressed between the sexes (that is what I think of when I read sexually-dimorphic transcriptomes) and other patterns that differ between males and females, such as gene inheritance or evolution. This happens again on line 583 which suggests the data in Figure 4 is somehow broken out by male- or female-biased expression, which I do not see.

>>> To make it more clear for the reader we changed the word "transcriptomes" to "regulatory evolution and expression inheritance", which is what is denoted in Fig. 4 and not sex-biased expression. That said, Fig. 6 does show those results broken down by sex-biased expression and also shows distinct patterns for males and females.

In the equation on line 908, one of the “Cbr" should be a “Cni” I think

>>> Done. Thanks!
